# Resequencing of the *Leishmania infantum* (strain JPCM5) genome and *de novo* assembly into 36 contigs

**DOI:** 10.1038/s41598-017-18374-y

**Published:** 2017-12-22

**Authors:** Sandra González-de la Fuente, Ramón Peiró-Pastor, Alberto Rastrojo, Javier Moreno, Fernando Carrasco-Ramiro, Jose M. Requena, Begoña Aguado

**Affiliations:** 1 Centro de Biología Molecular “Severo Ochoa” (CSIC-UAM), Campus de Excelencia Internacional (CEI) UAM+CSIC, Universidad Autónoma de Madrid, Madrid, Spain; 2 World Health Organization Collaborating Centre for Leishmaniasis, Laboratory of Reference and Research in Parasitology, Centro Nacional de Microbiologia, Instituto de Salud Carlos III, Madrid, Spain; 3 Red de Investigación Colaborativa en Enfermedades Tropicales (RICET), ISCIII, Madrid, Spain

## Abstract

*Leishmania* parasites are the causative of leishmaniasis, a group of potentially fatal human diseases. Control strategies for leishmaniasis can be enhanced by genome based investigations. The publication in 2005 of the *Leishmania major* genome sequence, and two years later the genomes for the species *Leishmania braziliensis* and *Leishmania infantum* were major milestones. Since then, the *L. infantum* genome, although highly fragmented and incomplete, has been used widely as the reference genome to address whole transcriptomics and proteomics studies. Here, we report the sequencing of the *L. infantum* genome by two NGS methodologies and, as a result, the complete genome assembly on 36 contigs (chromosomes). Regarding the present *L. infantum* genome-draft, 495 new genes have been annotated, a hundred have been corrected and 75 previous annotated genes have been discontinued. These changes are not only the result of an increase in the genome size, but a significant contribution derives from the existence of a large number of incorrectly assembled regions in current chromosomal scaffolds. Furthermore, an improved assembly of tandemly repeated genes has been obtained. All these analyses support that the *de novo* assembled *L. infantum* genome represents a robust assembly and should replace the currently available in the databases.

## Introduction

Protists of the genus *Leishmania* belong to the order Trypanosomatida, an early-branching line from the eukaryotic tree^1^. Many species of the genus are highly pathogenic for humans and other mammals, causing several clinical manifestations that are globally known as leishmaniasis. These pathogenic *Leishmania* species are transmitted by phlebotomine sand flies^2^. Although it is not absolute, there exists an association between the clinical forms of leishmaniasis and the infecting *Leishmania* species^3^. Thus, the clinical spectrum of leishmaniasis encompasses subclinical (asymptomatic) infections, self-healing cutaneous lesions, and disseminated forms (diffuse cutaneous, mucosal, or visceral leishmaniasis). *Leishmania major* is the prototypical species associated with cutaneous leishmaniasis in the Old World, mucosal affections (also known as mucocutaneous leishmaniasis) are hallmarks of *Leishmania braziliensis* infection, whereas *Leishmania donovani* and *Leishmania infantum* are the causative agents of visceral leishmaniasis (VL). The latter species are closely related, according to molecular genetic criteria^4^, even though they are found in different geographical regions: *L. donovani* is the primary cause of VL in the Indian subcontinent and East Africa, and *L. infantum* is the causative agent of VL in the Mediterranean basin, the Middle East, and Latin America^5^.

The medical relevance, together with the peculiarities in molecular mechanisms and biological structures present in this group of microorganisms^6^, justified efforts leading to determine their precise genome sequence. *L. major* was the first species of them to have its genome sequenced^7^, and it provided the model/template for subsequent genomic analyses of other *Leishmania* species. Afterwards, in 2007, the sequences of the *L. braziliensis* and *L. infantum* genomes were published^8^. During the last decade, the extraordinary progress in genome sequencing technologies^9^, together with a significant reduction of sequencing costs, has speeded up the development of sequencing projects. As a positive consequence of this, an exponential increase in the number and diversity of sequenced genomes is taking place. In particular, based on the use of these new technologies, genomic drafts for several *Leishmania* species (and strains) have been published^10–18^ and/or are publically available in databases (*e.g*., TriTrypDB.org). Nevertheless, although these genomic sequences are providing valuable data, the *L. major* (Friedlin strain) genome, decoded ten years ago using classical Sanger sequencing, continues to be the best assembled genome for the genus *Leishmania*. In fact, given the remarkable degree of synteny observed between the genomes of the different *Leishmania* species, the *L. major* genome is being used as the reference for building the chromosomal scaffolds of the other *Leishmania* species. However, apart from the *L. major* (Friedlin) genome, the rest of *Leishmania* genomes sequenced to date must be considered as draft assemblies, taking into account that they have been assembled in a number of contigs (between 562 and 10305) that vastly outnumbers the haploid set of chromosomes (34–36, depending on the *Leishmania* species^19^). The existence of a large number of repetitive DNA sequences, which are scattered along the *Leishmania* genomes^20–22^, prevents the complete assembly of the *Leishmania* chromosomes when using short-read sequencing approaches. Another challenging issue to resolve during the genomic assemblies is the precise determination of the gene copy number in loci consisting of multiple tandemly arranged identical genes, a common feature of the *Leishmania* gene organization^23^. In fact, as we recently reported, these issues were the cause of assembly collapses affecting seven genomic regions that were missed at the time of the *L. major* (Friedlin) genome assembly^24^.

For sequencing the genome of the *L. infantum* species, the JPCM5 strain (MCAN/ES/98/LLM-724) was selected^8^. This strain was first isolated in Madrid (Spain) from a naturally infected dog that developed VL^25^. This strain was cryopreserved at the Centro Nacional de Microbiología (Instituto de Salud Carlos III, Madrid, Spain) and distributed to different laboratories around the world. This strain showed a high degree of virulence when assayed for experimental infections of dogs^26^. Furthermore, it was found to be suitable for genetic manipulations, and importantly that the laboratory-modified parasites retained their virulence^27^. All these studies supported the selection of this strain for the generation of the first genome draft of a viscerotropic *Leishmania* species^8^. Genome sequences were produced by the whole-genome shotgun method, achieving a mean coverage of five-fold. The sequences reads were assembled into 562 contigs, which finally were grouped into 36 chromosomal scaffolds using as reference the *L. major* (Friedlin) genome^7^. At that time, the authors estimated that the missing genomic regions would account for at least 150,519 bases^8^.

Here, due to the clinical relevance of *L. infantum* together with the fact that this species is a widely used model for molecular biology purposes, we undertook the aim to improve its genome assembly, currently formed by several hundreds of contigs. For that objective, we sequenced the *L. infantum* (JPCM5 strain) genome using two different platforms, the Pacific Biosciences (PacBio) technology, used to produce sequencing reads of 10–15 kb in lengths, and the Illumina technology to generate paired-end short-reads. As a result, we are providing one of the most comprehensive reference genomes available, having a quality at least comparable to the *L. major* (Friedlin) genome assembly, and clearly better than the current *L. infantum* (JPCM5) one.

## Results and Discussion

### Illumina sequencing and assembly results

Total DNA isolated from *L. infantum* (JPCM5) promastigotes was sequenced with the Illumina high-throughput sequencing technology. A total of 56,327,604 paired-end 126 bp sequence reads with an average insert size of 310 bp were obtained. Thus, according to the estimated genome size of this *Leishmania* strain (32,134,935 bp^8^), these reads would correspond to a sequencing coverage above 400×. A *de novo* assembly from these data was tried using several assemblers, and the best results were achieved with the CLC Genomics Workbench software (CLC Bio; version 5.0). After several refinements, the final assembly yielded 1874 scaffolds with a size of the longest contig of 363,515-bp and a total genome size of 31,179,733-bp. Thus, in spite of having a better sequence-depth, the assembly did not substantially improve the current draft for the *L. infantum* genome^8^. *L. infantum* and other *Leishmania* species contain many repetitive sequences, with sizes around 500–600 bp, scattered throughout the genome^20–22^. The high sequence identity shared by some of these repeated elements is a cause of conflicts for assemblers^28^ when using short reads as those generated by Illumina and other sequencing platforms^9^. A strategy often followed to circumvent this drawback consists in ordering the contigs into scaffolds using a well-assembled genome from a related organism as the reference. In this context, the *L. major* (Friendly) genome has been used to assemble most of the *Leishmania* genomes currently available at the TriTryDB database. However, this is a risky strategy that could induce misassembling in divergent regions. In addition, a large number of tandemly repeated, multi-copy genes exist in the *Leishmania* genomes^23^, and to determine the exact copy number of those genes is another challenge to resolve, when working with short reads, since repetitive sequence regions tend to collapse into a single copy if no nucleotide differences exists to distinguish among the gene copies^28^. On the other hand, to define the copy number by comparison with a reference, assuming that the copy number is conserved among different *Leishmania* species, is not accurate.

### PacBio sequencing is a key technology for Leishmania genome assembly

As short-read paired-end Illumina technology resulted insufficient to achieve a full assembly of the *L. infantum* genome, it was decided to generate long-read sequences based on the single-molecule real-time (SMRT) sequencing technology developed by Pacific Biosciences (PacBio^29^). A total of 311,471 reads with an average length of 11700- bp were obtained. A coverage of around 100× was estimated, taking into account the size of the *L. infantum* genome currently available at GeneDB. Interestingly, *de novo* genome assembly using the RS_HGAP_Assembly.3 protocol of the Pacific Biosciences SMRT Analysis Software v2.3.0^30^ yielded 85 contigs, a number closer to the real number of *L. infantum* chromosomes, i.e. 36^19^. Of these, 41 were discarded as “spurious or artefactual”, some of which corresponded to maxicircle sequences and others were short sequences showing low read coverage and high sequence similarity with regions present in the long contigs (see Materials and Methods for further details).

**Figure 1:**
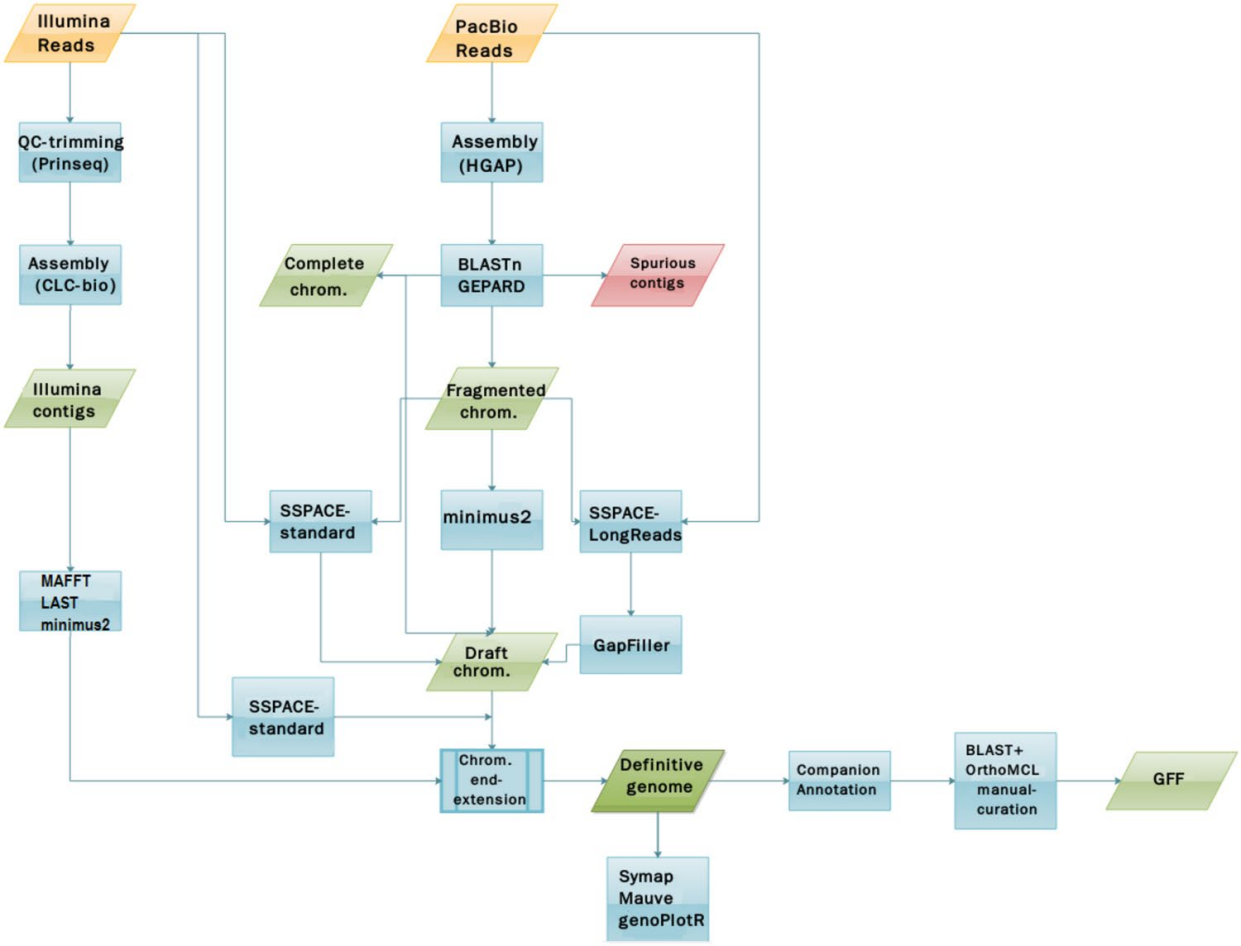
Schematic overview of the workflow leading to the *L. infantum* genome assembly. Input files (Raw Reads) are represented as yellow rhomboids. All the different software and processes are shown in blue boxes. Output files are represented in green rhomboids. Discarded data are shown in red rhomboids. See Materials and Methods section for additional details.

From the 44 remaining contigs, with the aid of the contigs generated from the Illumina reads data (see above) and using different assemblers and other bioinformatics tools, it was possible to join some of them, and finally reducing the total number of contigs to 36. Chromosomes 7, 12, 15, 22, 26, 28, 33 and 35 resulted by joining two contigs, but the rest of chromosomes were directly assembled from PacBio reads as a sole contig. Figure 1 shows the pipeline followed to achieve the final assembly (further technical details have been provided in the Materials and Methods section).

**Figure 2:**
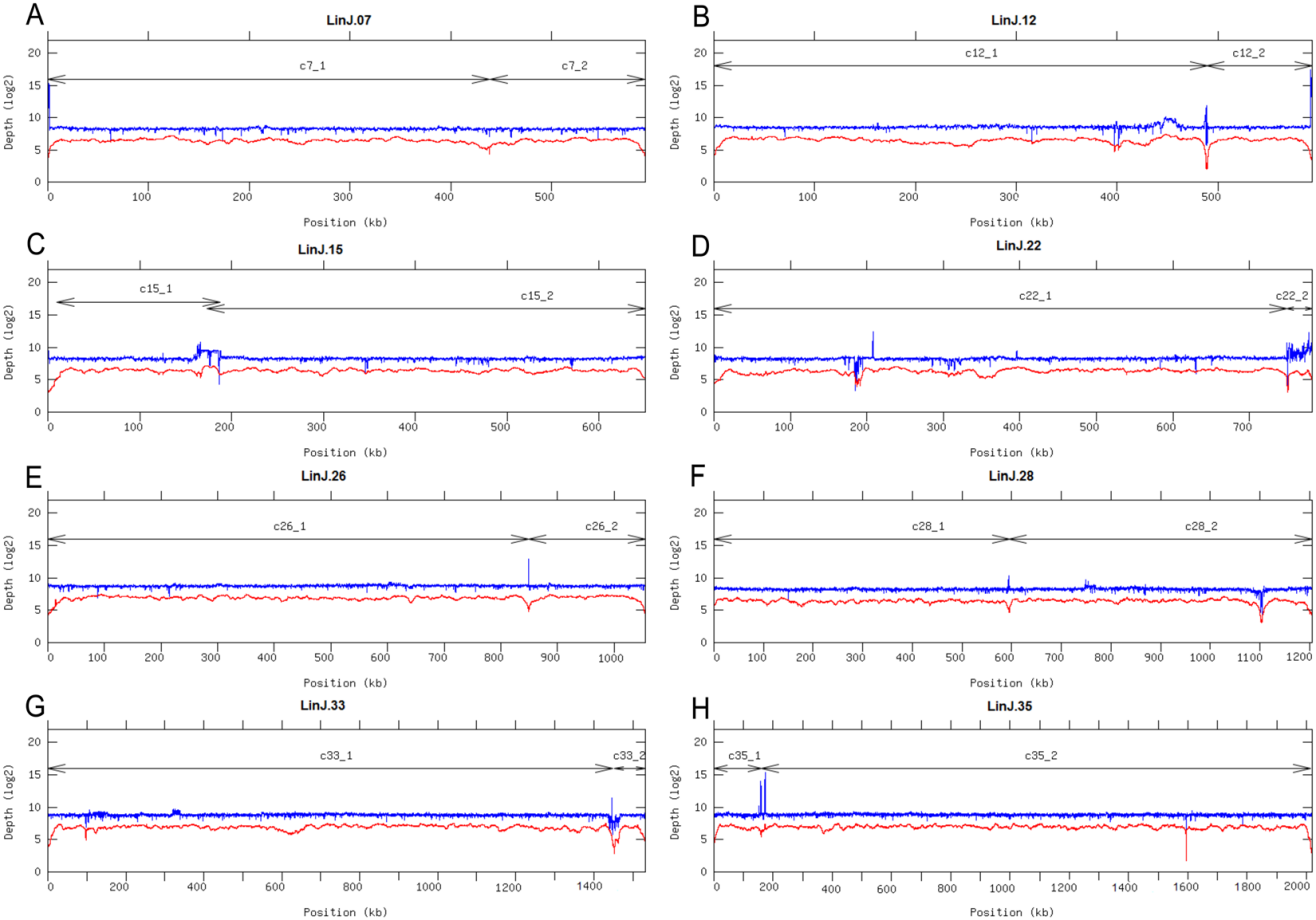
Read-depth analysis along the chromosomes formed by the fusion of two PacBio-assembled contigs. Coverage was determined by sliding window analysis (bin 200 pb) with either Illumina (in blue) or PacBio (in red) reads, along chromosomes 7, 12, 15, 22, 26, 28, 33 and 35. The sizes of the contigs are shown by lines with arrow-heads. Chromosomes 7 (panel A) and 35 (panel H) were joined by the SSPACE-standard tool. Chromosomes 12, 15, 22, 26 and 28 (panels B–F) were joined using the minimus 2 assembler. Finally, chromosome 33 (panel G) was joined by the SSPACE-LongRead tool.

The accuracy of the assembled 36 contigs was assessed by alignment of both Illumina and PacBio reads to the assembled chromosomes. The read distribution on those chromosomes formed by two PacBio-derived contigs is shown in Fig. 2. It was observed a homogeneous distribution of reads along the joined regions, suggesting that the assembly was properly performed.

In summary, our results demonstrated that PacBio sequencing is appropriate for achieving an effective assembly of *Leishmania* genomes, but Illumina sequencing was relevant for accurately joining some contigs and for extending the chromosomal ends of chromosomes (Fig. 1).

### Improvements in the *L. infantum* genome assembly

Table 1 summarizes major changes introduced in the new genome assembly regarding the previous one^8^. The total size of the genome (32,802,969-bp) has increased in 680,199-bp, and the number of undetermined nucleotides has been reduced from 20,399 in the current genome to zero in the new assembly. In addition, the chromosome 0, a chromosome created by the artificial joining of 34 genomic regions that could not be assigned with certainty to any of the 36 chromosomes, has disappeared after the *de novo* assembly reported here.

**Table 1:** Comparison of *L. infantum* genome assemblies.

**Features**	***L. infantum*** **-Ref** ^8^	***L. infantum*** **-New**
Number of chromosomes (scaffolds)	37	36
Number of contigs	562	36
Annotated genes	8195	8796
Number of gaps	470	0
Number of Ns	20399	0
Genome size (bp)	32122770	32802969
Coverage mean	5 × (Sanger sequencing)	370 × (Illumina)/97.43 × (PacBio)

**Figure 3:**
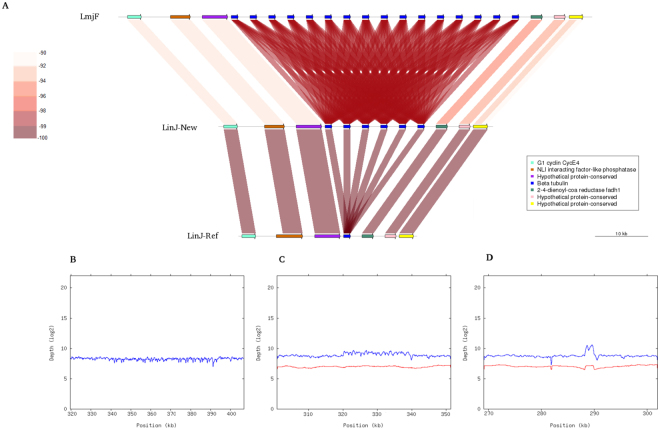
Gene copy number in the beta-tubulin locus at the chromosome 33. Panel (A): Genomic structure of the region containing the beta-tubulin locus in the *L. major* Friedlin genome (LmjF), in the *L. infantum* genome assembled in this work (LinJ-New) and in the current *L. infantum* assembly (LinJ-Ref).The identity percentage of the BLAST alignment (using the tab –format output of BLAST) is shown by shading with brown hue (scale at top left ranges from 90 to 100% of sequence identity). (**B**) Distribution of *L. major* Illumina reads (unpublished laboratory data) along the beta-tubulin locus in the *L. major* current genome (GeneDB.org). (**C**) Distribution of *L. infantum* sequence-reads (Illumina in blue and PacBio in red) along the beta-tubulin genomic region using as reference the *L. infantum* genome assembled in this work. (**D**) Distribution of *L. infantum* reads (Illumina in blue and PacBio in red) along the region containing the beta-tubulin locus in current *L. infantum* genome (version 9; Tritryp.org).

**Figure 4:**
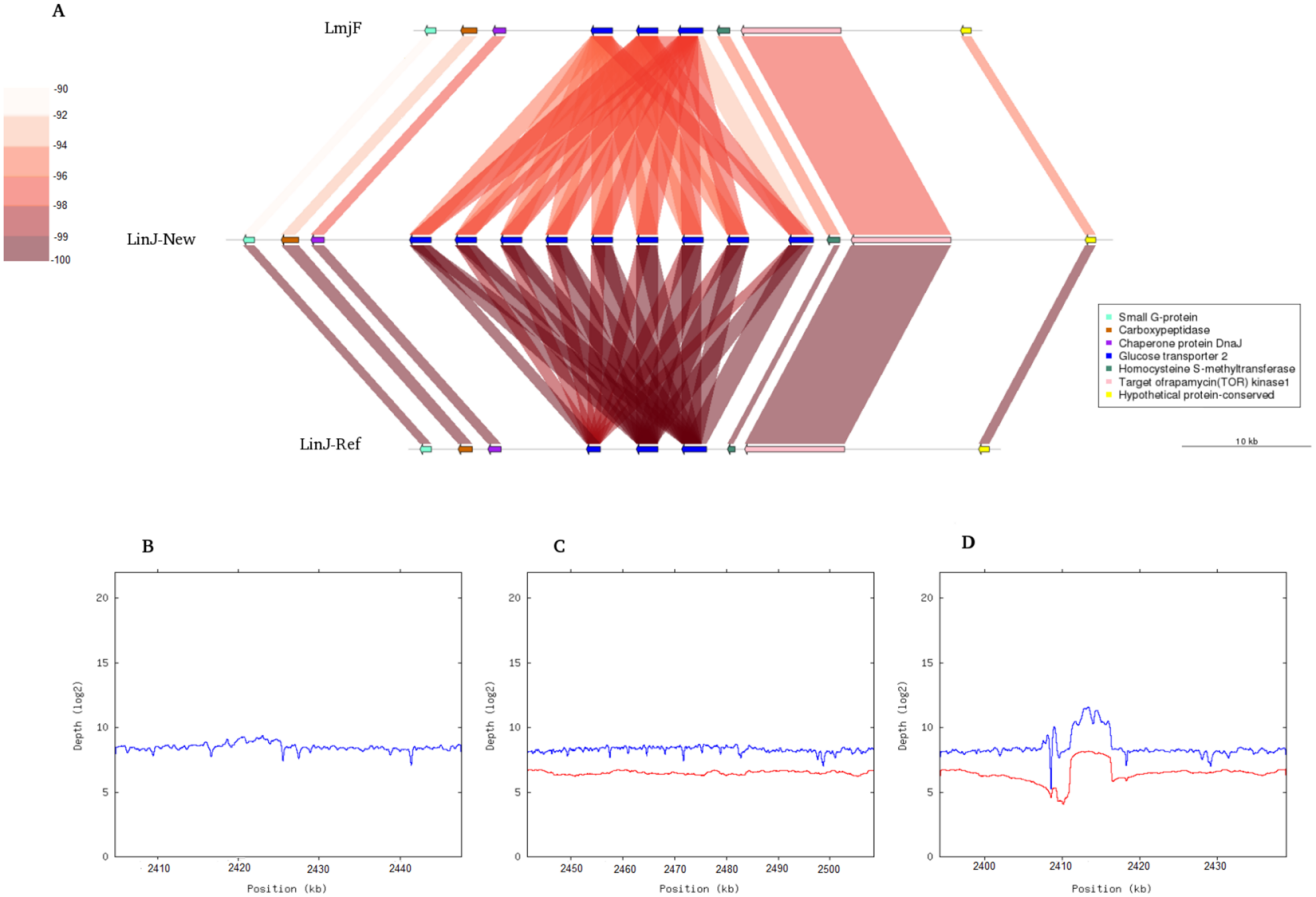
Gene copy number in the glucose transporter 2 locus. (**A**) Genomic structure of the region containing the glucose transporter 2 locus in the *L. major* Friedlin genome (LmjF), in the *L. infantum* genome assembled in this work (LinJ-New) and in the current *L. infantum* assembly (LinJ-Ref). See legend to Fig. 3 for the meaning of color codes. (**B**) Distribution of *L. major* Illumina reads along the glucose transporter 2 region in the *L. major* current genome (GeneDB). (**C**) Distribution of *L. infantum* sequence-reads (Illumina in blue and PacBio in red) along the glucose transporter 2 genomic region using as reference the *L. infantum* genome assembled in this work. (**D**) Distribution of *L. infantum* reads (Illumina in blue and PacBio in red) along the region containing the glucose transporter 2 locus in current *L. infantum* genome (version 9; Tritryp.org).

In agreement with the increase in the genomic size, the number of annotated genes has increased in 601, growing from 8195 (ref.^8^) to 8796 (including also non-coding RNAs), in the *de novo* assembly reported here. In particular, we have identified 495 new protein-coding genes (Table 2). An important fraction of these new genes corresponds to tandemly repeated genes that collapsed into one or two copies in the assembly of the current reference genome due to the relatively short size of the cloned fragments (4-kb or lower) and the sequence lengths (600–800 bp^8^). Thus, a significant increase in the number of annotated genes has been determined in the loci coding for cysteine peptidase B (CPB; LinJ.08), ATG8 (LinJ.09 and LinJ.19), GP63 – leishmanolysin (LinJ.10), alpha tubulin (LinJ.13), elongation factor 1-alpha (LinJ.17), glycerol uptake protein (LinJ.19), calpain-like cysteine peptidase (LinJ.27), putative Snf7 (LinJ.27), HSP70 (LinJ.28), paraflagellar rod protein (LinJ.29), 3-ketoacyl-CoA thiolase-like protein (LinJ.31), HSP83/90 (LinJ.33), beta tubulin (LinJ.33; Fig. 3), flagellar member 8 (LinJ.33), amastin-like surface protein (LinJ.34), 60 S ribosomal protein L2 (LinJ.35), glucose transporter 2 (LinJ.36; Fig. 4), and several hypothetical proteins (LinJ.21, LinJ.22, LinJ.27 and LinJ.31). As *Leishmania* parasites lack transcriptional control for gene expression, the presence of tandemly organized copies of the same gene has been suggested to serve as a way of increasing the expression level of critical proteins^31^.

**Table 2:** Size of chromosomes and annotated genes in the new assembly of *L. infantum* (JPCM5) genome.

**Chromosome**	**Size-Ref**	**Size-New assembly**	**Annotated genes**	**New genes**
LinJ.00	197816	—		
LinJ.01	277951 (202)	278268	86	1
LinJ.02	334113 (6)	356299	79	7
LinJ.03	382367 (203)	389660	101	3
LinJ.04	475338 (707)	466506	129	4
LinJ.05	449024 (306)	467711	150	3
LinJ.06	523352	525234	143	7
LinJ.07	592382 (1321)	592865	136	6
LinJ.08	495393 (5)	515744	130	13
LinJ.09	572115 (6)	581921	184	10
LinJ.10	547235 (518)	588571	166	18
LinJ.11	575792 (204)	568610	152	6
LinJ.12	568477 (1508)	593479	129	18
LinJ.13	645761 (814)	659809	176	17
LinJ.14	639279 (711)	656122	168	10
LinJ.15	617636 (825)	650312	190	26
LinJ.16	698903 (907)	688194	181	10
LinJ.17	667340 (805)	690898	173	14
LinJ.18	720194 (412)	720421	177	8
LinJ.19	742501 (13)	706116	184	17
LinJ.20	732590 (503)	731246	183	5
LinJ.21	759899 (407)	764851	240	6
LinJ.22	659512 (656)	782138	183	18
LinJ.23	774004	786675	220	10
LinJ.24	867075 (53)	863800	252	2
LinJ.25	886912 (706)	895070	273	14
LinJ.26	1050165 (1109)	1055294	282	8
LinJ.27	1043947 (531)	1175405	300	19
LinJ.28	1163438 (64)	1205018	338	19
LinJ.29	1221905 (713)	1272412	317	16
LinJ.30	1365115 (201)	1353282	389	9
LinJ.31	1468864 (708)	1529233	370	31
LinJ.32	1547509	1544753	427	17
LinJ.33	1448148 (830)	1532280	381	36
LinJ.34	1668239 (1697)	1852060	481	40
LinJ.35	2068523 (1720)	2019666	548	15
LinJ.36	2673956 (1028)	2743046	778	32
Genome	32122770 (20399)	32802969	8796	495

For comparisons, current version (2015-12-07) of *L. infantum* genome (Ref), available at TriTrypDB, was used. This version contains a chromosome LinJ.00 that is formed by 34 genomic regions of uncertain chromosomal location. The number of undetermined nucleotides in the Ref genome is indicated in brackets.

Figures 3 and 4 illustrate the corrections introduced regarding the copy number in the loci for beta-tubulin and glucose transporter 2, respectively. For comparison, it is shown the structure of these loci in *L. major* (Friedlin) and in the current reference *L. infantum* (GeneDB.org) genomes, together with the one derived from the new assembly described here (panels A). The homogeneous distribution of reads (either from Illumina or PacBio data) along the new assembled genome (panels C) contrast with the irregular distribution found when the reference genome (GeneDB.org) was used for reads-alignment (panels D). In the current *L. infantum* assembly (LinJ-Ref), a sole beta-tubulin gene was assembled, whereas six genes were assembled in the *L. infantum* genome reported in this work (LinJ-New). Noticeably, 16 beta-tubulin genes are assembled in the *L. major* (Friendly) genome (see Fig. 3). By contrast, for the glucose transporter 2 the current (LinJ-Ref) and *L. major* genomes show three copies, but in the new assembly (LinJ-New) six copies were detected (see Fig. 4). For most of the loci containing tandemly repeated genes, it is likely that the correct number of genes has been assembled, as reads coverage was found to be quite homogeneous along the 36 chromosomes of the new assembled *L. infantum* genome (Fig. 2, and data not shown). The sole exception is the rDNA locus, for which our assembly contains three tandemly linked copies of the 18 S rRNA- 5.8 S rRNA-24S α/β rRNA unit; however, by measuring the mean read depth of this genomic region and normalization with the mean read coverage of chromosome 27, it was determined that the total number of rDNA units would be between 6 and 9 copies. The size of repetition unit (~10.5 Kbp) justifies that the PacBio reads also collapsed during the assembly of the rDNA locus. In current *L. infantum* genome database (GeneDB.org), no annotation on the rDNA locus exists; nevertheless, visual analysis of the assembled sequence indicated that only an rDNA unit was assembled.

### Nomenclature of the new assembled genome and synteny analysis

Among the new genes identified in the *de novo* assembled genome (Table 2) there are some genes that were not annotated in current databases because the corresponding genomic regions were missed. In addition, the new genome sequence has allowed to complete partial gene sequences and correct some sequence uncertainties (Ns) or errors. Current ID nomenclature of *L. infantum* genes (GeneDB.org) has been maintained as much as possible, as it is widely used and there are many publications having relevant data referring to that nomenclature. Hence, the former ID names have been kept even when some genes were found to be located at a different chromosome in the new assembly, regarding current reference genome. When new genes were needed to be named, the same nomenclature rules were used, and intercalated ID numbers were assigned to name these new genes. Finally, a total of 75 previously annotated genes have been excluded from the annotation of the new *L. infantum* genome. Some of the eliminated ID names corresponded to genes that were annotated as tandem gene duplications, but such gene duplications were not found in the new assembly. Other previous annotations were based on genomic regions that were bound in an artefactual manner (see below). In summary, we have tried to maintain as much as possible the former gene IDs, even when the ORFs had to be partially corrected. For a few cases, the former IDs were eliminated to avoid confusion, as the new ORFs were very different to the previously annotated.

**Figure 5:**
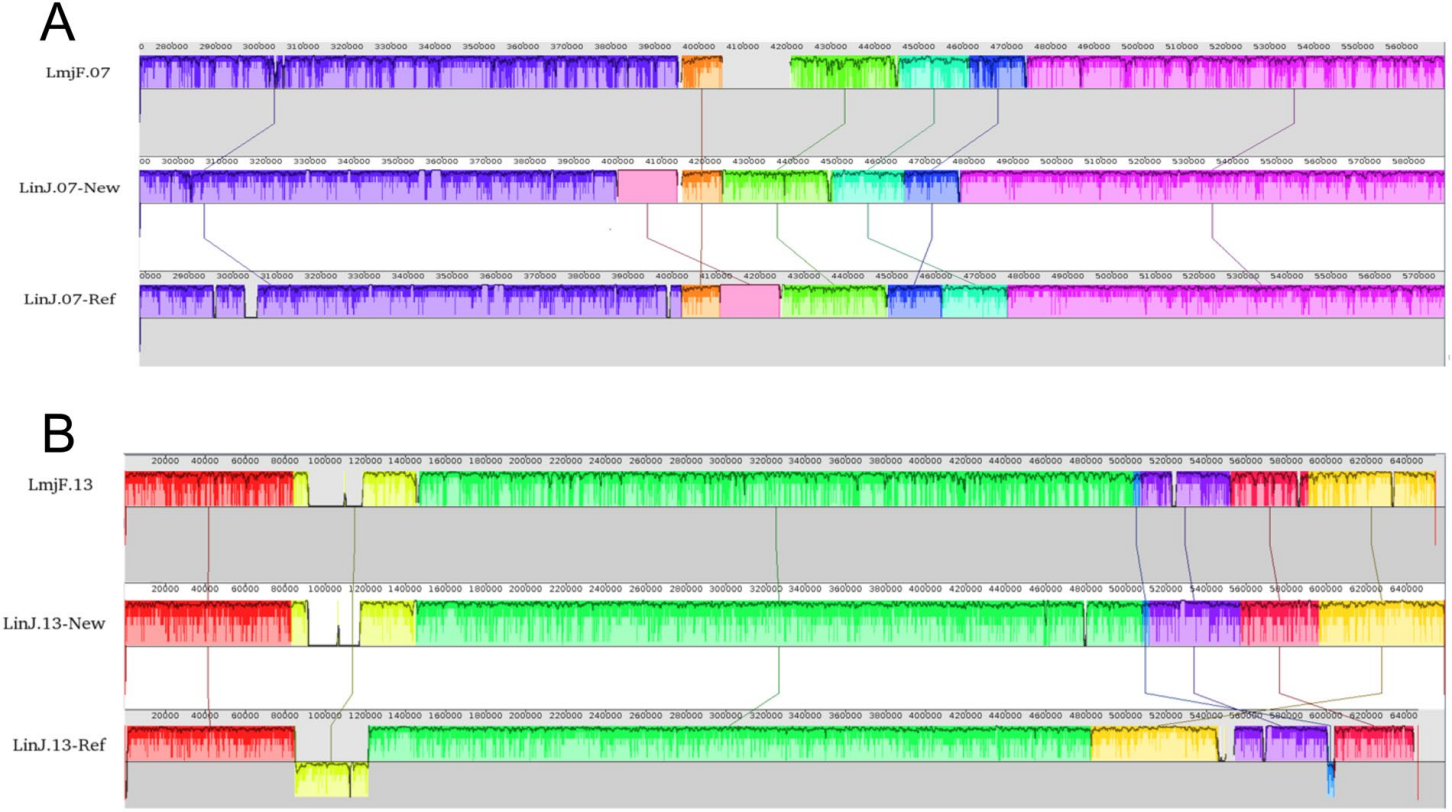
Schematic illustration of mis-assembled regions in the current *L. infantum* genome. Synteny blocks, represented by different colors, in chromosome 7 (panel A) and chromosome 13 (panel B) after pair-wise comparisons between the *L. major* Friedlin genome (top), the *L. infantum* newly assembled genome (middle) and *L. infantum* reference genome (bottom). Pairwise alignments were generated by the progressive MAUVE algorithm, which uses color codes to depict blocks of conserved regions. Sections located underneath the x-axis show inversion events.

On the other hand, a comparison between the current genome and the *de novo* annotated one highlighted important reorganizations in most of the chromosomes. In Fig. 5, two illustrative examples, affecting chromosomes 7 and 13, are shown. In these cases, the synteny of the homologous chromosomes in the *L. major* (Friedlin) genome is also shown. Remarkably, the *de novo* assembled genome is more syntenic when compared to the *L. major* assembly than when compared to the *L. infantum* reference genome, further supporting the view that some of these segments were incorrectly placed during the current *L. infantum* genome assembly^8^. In summary, all these analyses support the conclusion that the *de novo* assembled *L. infantum* genome described in this work represents a robust assembly. Full synteny maps comparing L. major (Friedlin) genome and the *L. infantum* genomes (new and reference) are provided in Supplementary Figures S1–S36.

### Conclusions

The availability of a robust genome sequence is a valuable resource for studies addressing whole-organism aspects following either genomics, transcriptomics or proteomics approaches. Advances in sequencing technologies have greatly facilitated genome sequencing tasks. However, genome sequencing data by themselves may have a limited utility unless they are adequately assembled in order to define the arrangement of genes and genome architecture. An enormous effort was invested to elucidate the genome structure and sequence of *L. major*, a milestone achieved in 2005. Genome comparison among the *Leishmania* species has shown a high degree of genome conservation in terms of both gene content and gene synteny across the genus. This finding led to the use of *L. major* (Friedlin) genome as a template to facilitate the assembly process of most of the *Leishmania* genomes reported afterwards. However, as demonstrated in this and other works^32^, that approach may lead to introduce assembly errors that would compromise future studies regarding gene content, gene models and genome architecture. Here, we present a *de novo* assembly of the *L. infantum* (JPCM5) genome based on sequence data derived from both long (PacBio) and short (Illumina) reads that yielded the expected 36 chromosomal-size contigs, without discontinuities and undetermined sequence (Ns), which are abundant in the current genome (GeneDB.org). Furthermore, this work is providing a methodological pipeline to obtain a full closed genome of a *Leishmania* species (or related kinetoplastids).

The new *L. infantum* genome sequence and annotations will be available at EBI databases, and also at the Leish-ESP web site (https://leishseq.neocities.org/). Moreover, the complete annotation of the new genome is provided in the Supplementary file 1. This complete annotation will considerably help to understanding the molecular processes underlying the biology of this malignant parasite and to the development of more effective control strategies.

## Methods

### Parasites and DNA isolation

The *L. infantum* reference strain, JPCM5 (MCAN/ES/98/LLM-724), was isolated by Dr J. Moreno’s group (WHO Collaborating Centre for Leishmaniasis, Centro Nacional de Microbiología, Instituto de Salud Carlos IIII, Madrid, Spain) from a dog suffering from visceral leishmaniasis. Promastigotes were cultured at 26 °C in RPMI 1640 medium (Sigma-Aldrich), supplemented with 20% heat-inactivated foetal calf serum (Sigma-Aldrich). Genomic DNA was isolated following the classical phenol-chloroform-isoamyl alcohol extraction method as described previously^33^.

### Illumina sequencing and reads assembly

Library construction and paired-end library sequencing were performed at the Centro Nacional de Análisis Genómico (CNAG-CRG, Spain) using Illumina HiSeq. 2000 technology. A total of 56,327,604 paired-end, 126 bp sequence reads were generated.

PrinseqQuality (http://prinseq.sourceforge.net/) was applied to quality filtering/trimming of reads (cut-off value, 20), and only reads with length ≥60-nt were used. Reads were assembled using the CLC Genomics Workbench version 5.0 (CLC Bio).

### PacBio sequencing and de novo assembly

The single-molecule real-time (SMRT) sequencing technology developed by Pacific Biosciences (PacBio)^29^ was used for long reads sequencing. A total of 311,471 pre-filtered reads were generated on a PacBio RS II sequencing instrument. The sequencing service was provided by the Norwegian Sequencing Centre (www.sequencing.uio.no), a national technology platform hosted by the University of Oslo and supported by the “Functional Genomics” and “Infrastructure” programs of the Research Council of Norway and the Southeastern Regional Health Authorities.

*De novo* genome assembly was carried out following a hierarchical genome-assembly process (HGAP), using the RS_HGAP_Assembly-3 protocol included in the PacBio SMRT Analysis Software v2.3.0 (Ref.^30^), with default settings and considering 35 Mb as expected genome size. Quality trimming of PacBio reads was done by default as part of the HGAP pipeline (P_filter Module).

### Assembly refinements

The 85 contigs, initially assembled by HGAP from the PacBio reads, were reanalyzed in order to discard those having a disproportionately low coverage (<40x) or short length (<15-Kb). Hence, 41 of those contigs were found to represent “spurious or artefactual” contigs, and were consequently discarded. After filtering, a total of 44 contigs were selected as *bona fide* genomic sequences. Twenty-eight of these contigs were found to correspond to complete chromosomes. For accurately assembling of the rest of chromosomes, different software packages and approaches were used^34^. Firstly, the Illumina paired sequencing reads were used to assess the possibility of joining some contigs by the SSPACE tool^35^. This approach allowed the complete assembly of chromosomes 7 and 35. The accuracy of these and the rest of assemblies was monitored by alignment of sequencing reads and its visualization by IGV^36^. Contigs belonging to the chromosomes 12, 15, 22, 26 and 28, were joined by the minimus2 assembler^37^, which uses an algorithm that calculates overlaps between contigs. On the other hand, the two contigs forming the chromosome 33 could be joined by means of the SSPACE-LongRead tool^38^, which selects and uses only the longest PacBio reads to construct a scaffolding. Finally, the gap size between pairs of contig was calculated (lower than 5-Kb in all cases) and closed with Gapfiller^39^, using the distance information derived from the paired-read data.

The contigs generated from the Illumina sequencing reads were aligned to the *de novo* assembled chromosomes using LAST aligner (http://last.cbrc.jp/). This allowed the identification of some Illumina contigs that aligned with the chromosomal ends but had overhanging sequences. In those cases, several tools were used to further extend the chromosomal ends. Thus, for chromosomes 4, 14 and 24, the optimal extension was attained with MAFFT multiple-aligner software^40^. For chromosomes 34 and 15, the best extension was obtained by minimus2. Finally, SSPACE-standard was found useful to extend a few nucleotides at the ends of the rest of chromosomes. A scheme of the complete pipeline is shown in Fig. 1.

Finally, a coverage analysis on the newly assembled chromosomes was performed using both Illumina and PacBio reads. Illumina reads were aligned by Bowtie2 (ref.^41^), and PacBio bax.h5 reads were aligned by pbalign (which uses the BLASR method^42^). Coverage analysis was done from each alignment along the 36 chromosomes using the GenomeCoverageBed tool (http://bedtools.readthedocs.io/en/latest/content/tools/genomecov.html). The graphical coverage plots files were generated with GNUPLOT (http://www.gnuplot.info/).

### Annotation of protein-coding genes and known non-coding RNAs

Bulk annotation of the assembled *L. infantum* genome was performed using Companion web server^43^, using the default settings and selecting the *L. major* (Friedlin strain) annotation as a reference. Given the importance of maintaining current *L. infantum* gene ID names, OrthoMCL^44^, BLAST searches were performed to assign correspondences between the Companion annotated genes and current *L. infantum* gene names (version 9, TriTrypDB.org). These additional data were combined with those provided by Companion annotation into a GFF3 file using an in-house Python script. Finally, the GFF3 file was manually curated to resolve ambiguous annotations and to name those new genes uncovered in the new assembled *L. infantum* genome. The complete annotation of the new genome (GFF3 file) is provided in the Supplementary file 1 in Excel format.

### Synteny analysis

Synteny was evaluated via SyMAP^45^ and progressive MAUVE^46^ algorithms using current *L. infantum* (v.9, GeneDB.org) and *L. major* ^24^ genomes as reference. Synteny graphs were prepared by genoPlotR^47^, and provided as Supplementary Figures S1–S36.

### Data availability

The Illumina paired ends reads (FASTQ) and PacBio bax.h5 reads of *L. infantum* (JPCM5 strain) generated for this study are available at The European Nucleotide Archive (ENA; http://www.ebi.ac.uk/ena/). Also, the assembled genome sequence and an annotation file were uploaded. All data have been deposited under the Study accession number PRJEB20254 and Study unique name: ena-STUDY-CBMSO-04-04-2017-10:39:08:689–498. The new *L. infantum* genome sequence will also be available at the Leish-ESP web site (https://leishseq.neocities.org/).

## Electronic supplementary material

Dataset 1

Supplementary Figures S1-S36
