# Supplementary material for: Resequencing of the Leishmania infantum (strain JPCM5) genome and de novo assembly into 36 contigs
Source: Sci Rep. 2017 Dec 22;7:18050. doi: 10.1038/s41598-017-18374-y (PMC5741766; doi:10.1038/s41598-017-18374-y)
Supplement: Supplementary file 2 — Supplementary Figures S1-S36 [file 41598_2017_18374_MOESM2_ESM.pdf]

Resequencing of the Leishmania infantum (strain JPCM5) genome and de novo assembly into 36 contigs

Sandra González-de la Fuente, Ramón Peiró-Pastor, Alberto Rastrojo, Javier Moreno, Fernando Carrasco-Ramiro, Jose M. Requena, Begoña Aguado

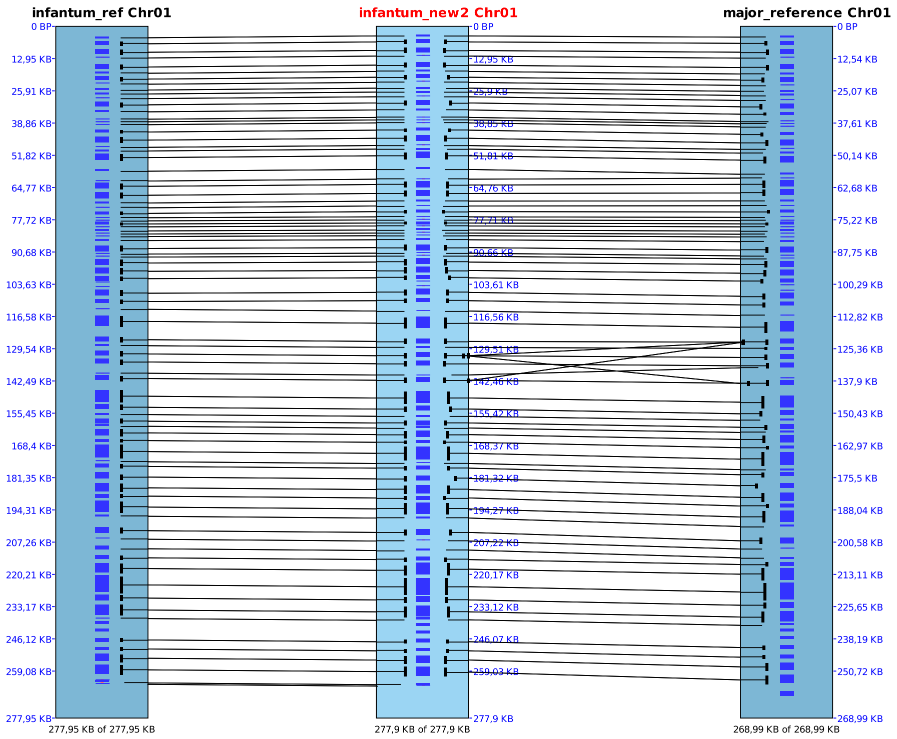

infantum_ref Chr01
infantum_new2 Chr01
major_reference Chr01
0 BP
12,95 KB
25,91 KB
38,86 KB
51,82 KB
64,77 KB
77,72 KB
90,68 KB
103,63 KB
116,58 KB
129,54 KB
142,49 KB
155,45 KB
168,4 KB
181,35 KB
194,31 KB
207,26 KB
220,21 KB
233,17 KB
246,12 KB
259,08 KB
277,95 KB
277,95 KB of 277,95 KB
0 BP
12,95 KB
25,9 KB
38,85 KB
51,81 KB
64,76 KB
77,71 KB
90,66 KB
103,61 KB
116,56 KB
129,51 KB
142,46 KB
155,42 KB
168,37 KB
181,32 KB
194,27 KB
207,22 KB
220,17 KB
233,12 KB
246,07 KB
259,03 KB
277,9 KB
277,9 KB of 277,9 KB
0 BP
12,54 KB
25,07 KB
37,61 KB
50,14 KB
62,68 KB
75,22 KB
87,75 KB
100,29 KB
112,82 KB
125,36 KB
137,9 KB
150,43 KB
162,97 KB
175,5 KB
188,04 KB
200,58 KB
213,11 KB
225,65 KB
238,19 KB
250,72 KB
268,99 KB
268,99 KB of 268,99 KB

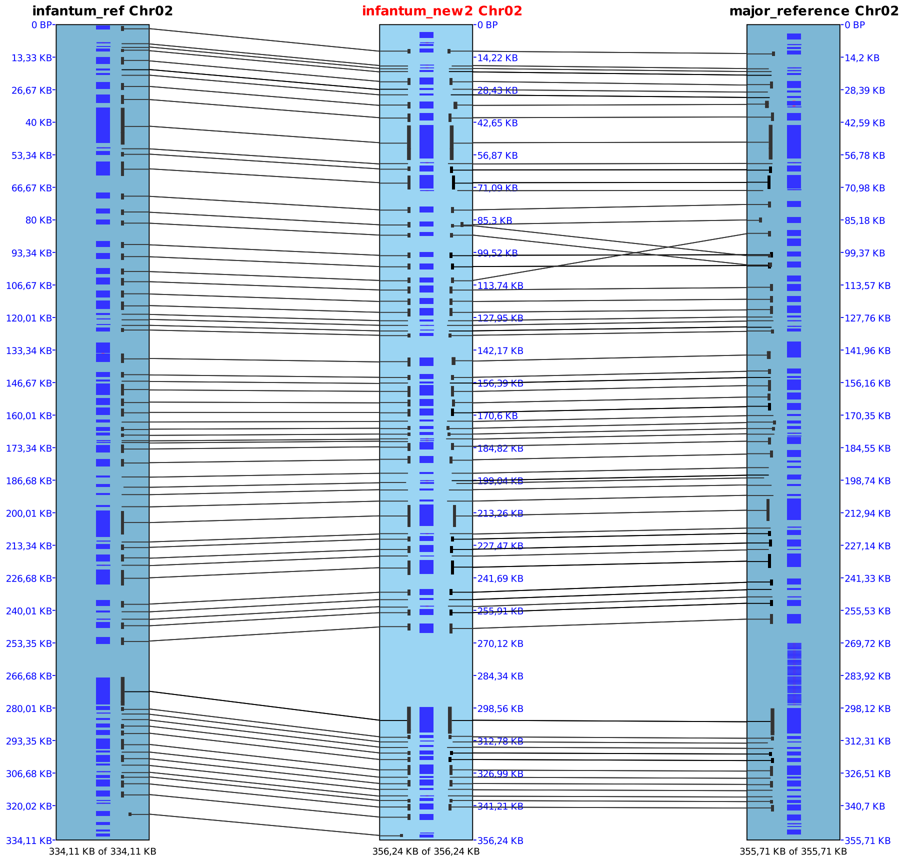

infantum_ref Chr02
infantum_new2 Chr02
major_reference Chr02
0 BP
13,33 KB
26,67 KB
40 KB
53,34 KB
66,67 KB
80 KB
93,34 KB
106,67 KB
120,01 KB
133,34 KB
146,67 KB
160,01 KB
173,34 KB
186,68 KB
200,01 KB
213,34 KB
226,68 KB
240,01 KB
253,35 KB
266,68 KB
280,01 KB
293,35 KB
306,68 KB
320,02 KB
334,11 KB
334,11 KB of 334,11 KB
0 BP
14,22 KB
28,43 KB
42,65 KB
56,87 KB
71,09 KB
85,3 KB
99,52 KB
113,74 KB
127,95 KB
142,17 KB
156,39 KB
170,6 KB
184,82 KB
199,04 KB
213,26 KB
227,47 KB
241,69 KB
255,91 KB
270,12 KB
284,34 KB
298,56 KB
312,78 KB
326,99 KB
341,21 KB
356,24 KB
356,24 KB of 356,24 KB
0 BP
14,2 KB
28,39 KB
42,59 KB
56,78 KB
70,98 KB
85,18 KB
99,37 KB
113,57 KB
127,76 KB
141,96 KB
156,16 KB
170,35 KB
184,55 KB
198,74 KB
212,94 KB
227,14 KB
241,33 KB
255,53 KB
269,72 KB
283,92 KB
298,12 KB
312,31 KB
326,51 KB
340,7 KB
355,71 KB
355,71 KB of 355,71 KB

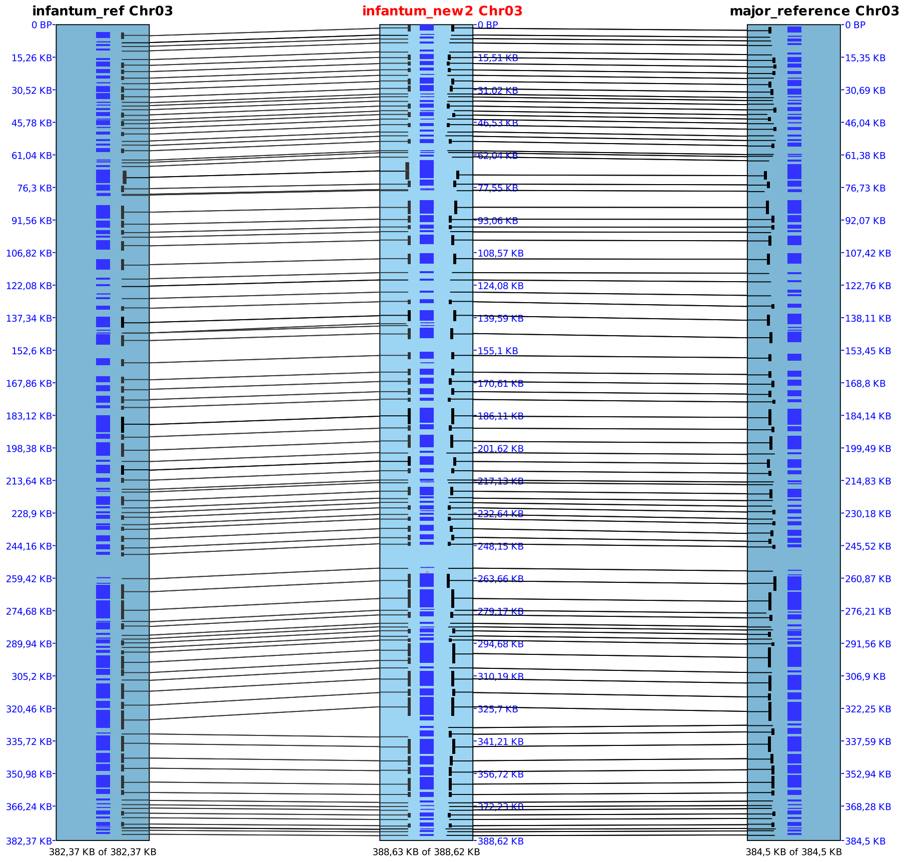
infantum_ref Chr03
infantum_new2 Chr03
major_reference Chr03
0 BP
15,26 KB
30,52 KB
45,78 KB
61,04 KB
76,3 KB
91,56 KB
106,82 KB
122,08 KB
137,34 KB
152,6 KB
167,86 KB
183,12 KB
198,38 KB
213,64 KB
228,9 KB
244,16 KB
259,42 KB
274,68 KB
289,94 KB
305,2 KB
320,46 KB
335,72 KB
350,98 KB
366,24 KB
382,37 KB
382,37 KB of 382,37 KB
0 BP
15,51 KB
31,02 KB
46,53 KB
62,04 KB
77,55 KB
93,06 KB
108,57 KB
124,08 KB
139,59 KB
155,1 KB
170,61 KB
186,11 KB
201,62 KB
217,13 KB
232,64 KB
248,15 KB
263,66 KB
279,17 KB
294,68 KB
310,19 KB
325,7 KB
341,21 KB
356,72 KB
372,23 KB
388,62 KB
388,63 KB of 388,62 KB
0 BP
15,35 KB
30,69 KB
46,04 KB
61,38 KB
76,73 KB
92,07 KB
107,42 KB
122,76 KB
138,11 KB
153,45 KB
168,8 KB
184,14 KB
199,49 KB
214,83 KB
230,18 KB
245,52 KB
260,87 KB
276,21 KB
291,56 KB
306,9 KB
322,25 KB
337,59 KB
352,94 KB
368,28 KB
384,5 KB
384,5 KB of 384,5 KB

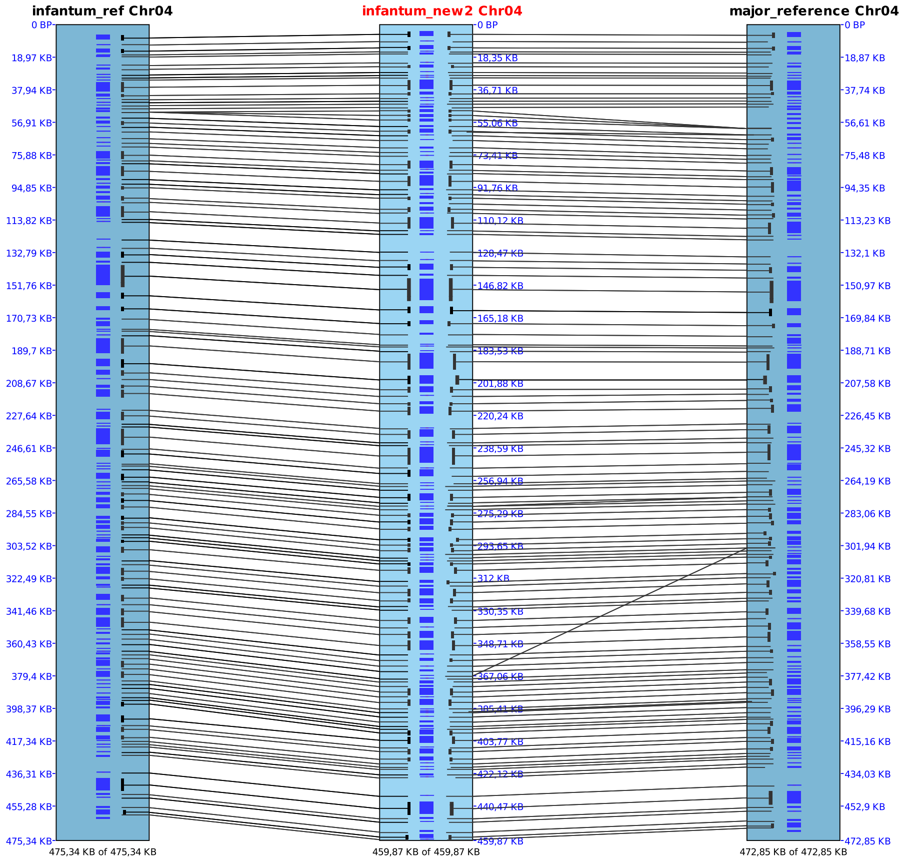

infantum_ref Chr04
infantum_new2 Chr04
major_reference Chr04
0 BP
18,97 KB
37,94 KB
56,91 KB
75,88 KB
94,85 KB
113,82 KB
132,79 KB
151,76 KB
170,73 KB
189,7 KB
208,67 KB
227,64 KB
246,61 KB
265,58 KB
284,55 KB
303,52 KB
322,49 KB
341,46 KB
360,43 KB
379,4 KB
398,37 KB
417,34 KB
436,31 KB
455,28 KB
475,34 KB
475,34 KB of 475,34 KB
0 BP
18,35 KB
36,71 KB
55,06 KB
73,41 KB
91,76 KB
110,12 KB
128,47 KB
146,82 KB
165,18 KB
183,53 KB
201,88 KB
220,24 KB
238,59 KB
256,94 KB
275,29 KB
293,65 KB
312 KB
330,35 KB
348,71 KB
367,06 KB
385,41 KB
403,77 KB
422,12 KB
440,47 KB
459,87 KB
459,87 KB of 459,87 KB
0 BP
18,87 KB
37,74 KB
56,61 KB
75,48 KB
94,35 KB
113,23 KB
132,1 KB
150,97 KB
169,84 KB
188,71 KB
207,58 KB
226,45 KB
245,32 KB
264,19 KB
283,06 KB
301,94 KB
320,81 KB
339,68 KB
358,55 KB
377,42 KB
396,29 KB
415,16 KB
434,03 KB
452,9 KB
472,85 KB
472,85 KB of 472,85 KB

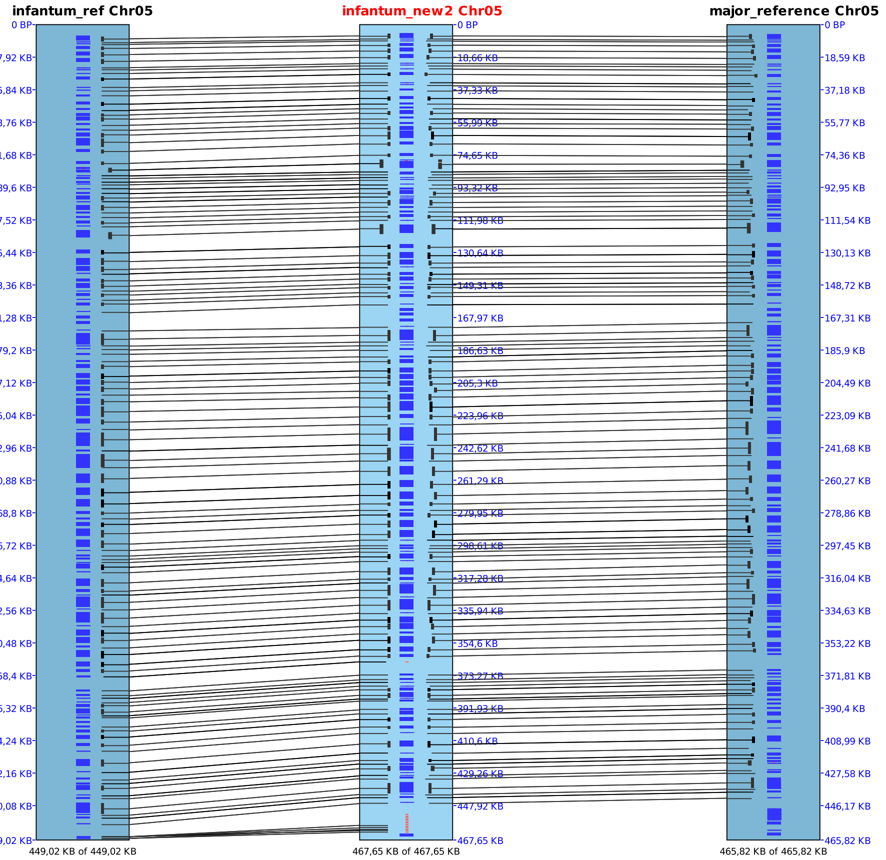

infantum_ref Chr05
infantum_new2 Chr05
major_reference Chr05
0 BP
17,92 KB
35,84 KB
53,76 KB
71,68 KB
89,6 KB
107,52 KB
125,44 KB
143,36 KB
161,28 KB
179,2 KB
197,12 KB
215,04 KB
232,96 KB
250,88 KB
268,8 KB
286,72 KB
304,64 KB
322,56 KB
340,48 KB
358,4 KB
376,32 KB
394,24 KB
412,16 KB
430,08 KB
449,02 KB
449,02 KB of 449,02 KB
0 BP
18,66 KB
37,33 KB
55,99 KB
74,65 KB
93,32 KB
111,98 KB
130,64 KB
149,31 KB
167,97 KB
186,63 KB
205,3 KB
223,96 KB
242,62 KB
261,29 KB
279,95 KB
298,61 KB
317,28 KB
335,94 KB
354,6 KB
373,27 KB
391,93 KB
410,6 KB
429,26 KB
447,92 KB
467,65 KB
467,65 KB of 467,65 KB
0 BP
18,59 KB
37,18 KB
55,77 KB
74,36 KB
92,95 KB
111,54 KB
130,13 KB
148,72 KB
167,31 KB
185,9 KB
204,49 KB
223,09 KB
241,68 KB
260,27 KB
278,86 KB
297,45 KB
316,04 KB
334,63 KB
353,22 KB
371,81 KB
390,4 KB
408,99 KB
427,58 KB
446,17 KB
465,82 KB
465,82 KB of 465,82 KB

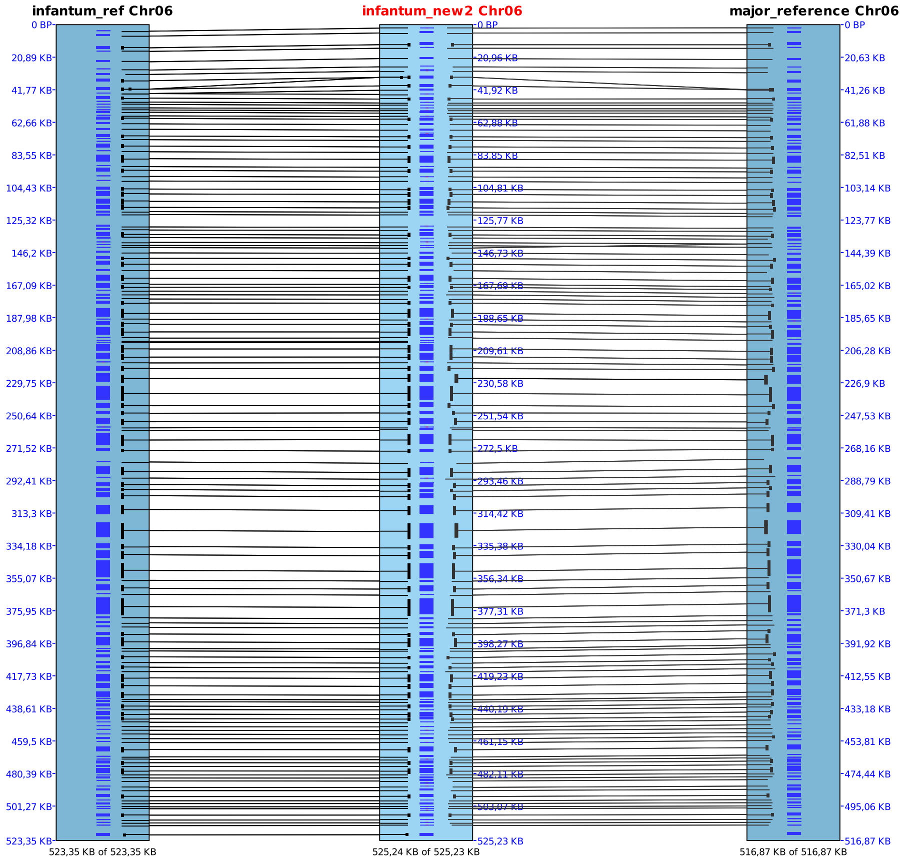
infantum_ref Chr06
infantum_new2 Chr06
major_reference Chr06
0 BP
20,89 KB
41,77 KB
62,66 KB
83,55 KB
104,43 KB
125,32 KB
146,2 KB
167,09 KB
187,98 KB
208,86 KB
229,75 KB
250,64 KB
271,52 KB
292,41 KB
313,3 KB
334,18 KB
355,07 KB
375,95 KB
396,84 KB
417,73 KB
438,61 KB
459,5 KB
480,39 KB
501,27 KB
523,35 KB
523,35 KB of 523,35 KB
0 BP
20,96 KB
41,92 KB
62,88 KB
83,85 KB
104,81 KB
125,77 KB
146,73 KB
167,69 KB
188,65 KB
209,61 KB
230,58 KB
251,54 KB
272,5 KB
293,46 KB
314,42 KB
335,38 KB
356,34 KB
377,31 KB
398,27 KB
419,23 KB
440,19 KB
461,15 KB
482,11 KB
503,07 KB
525,23 KB
525,24 KB of 525,23 KB
0 BP
20,63 KB
41,26 KB
61,88 KB
82,51 KB
103,14 KB
123,77 KB
144,39 KB
165,02 KB
185,65 KB
206,28 KB
226,9 KB
247,53 KB
268,16 KB
288,79 KB
309,41 KB
330,04 KB
350,67 KB
371,3 KB
391,92 KB
412,55 KB
433,18 KB
453,81 KB
474,44 KB
495,06 KB
516,87 KB
516,87 KB of 516,87 KB

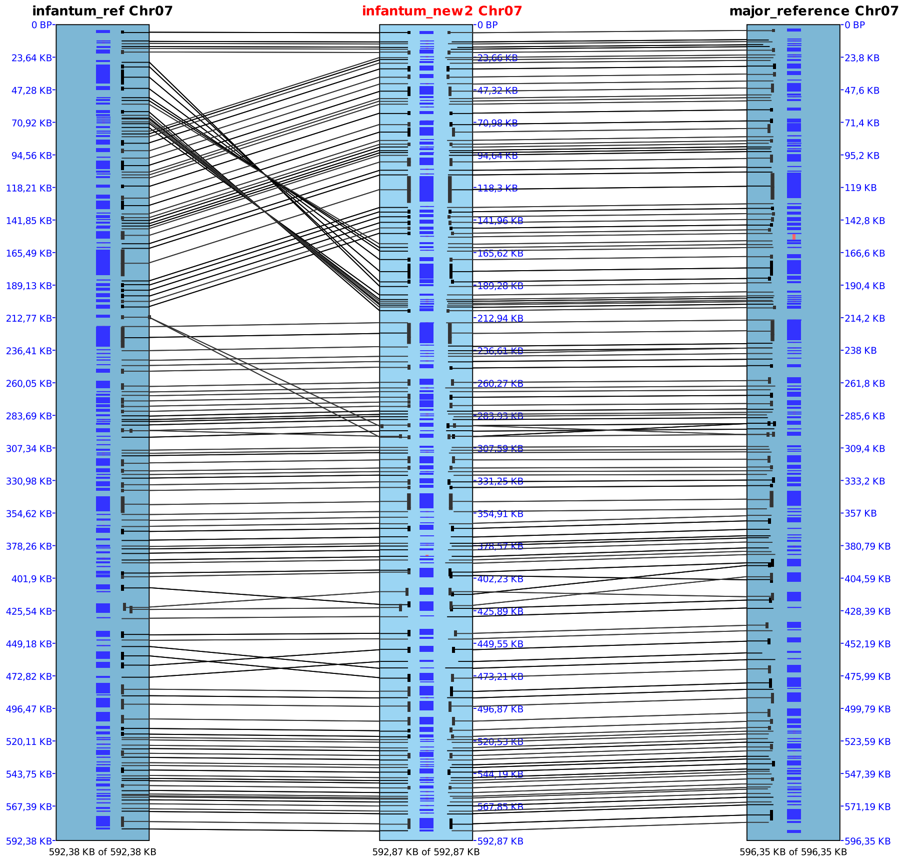
infantum_ref Chr07
infantum_new2 Chr07
major_reference Chr07
0 BP
23,64 KB
47,28 KB
70,92 KB
94,56 KB
118,21 KB
141,85 KB
165,49 KB
189,13 KB
212,77 KB
236,41 KB
260,05 KB
283,69 KB
307,34 KB
330,98 KB
354,62 KB
378,26 KB
401,9 KB
425,54 KB
449,18 KB
472,82 KB
496,47 KB
520,11 KB
543,75 KB
567,39 KB
592,38 KB
592,38 KB of 592,38 KB
0 BP
23,66 KB
47,32 KB
70,98 KB
94,64 KB
118,3 KB
141,96 KB
165,62 KB
189,28 KB
212,94 KB
236,61 KB
260,27 KB
283,93 KB
307,59 KB
331,25 KB
354,91 KB
378,57 KB
402,23 KB
425,89 KB
449,55 KB
473,21 KB
496,87 KB
520,53 KB
544,19 KB
567,85 KB
592,87 KB
592,87 KB of 592,87 KB
0 BP
23,8 KB
47,6 KB
71,4 KB
95,2 KB
119 KB
142,8 KB
166,6 KB
190,4 KB
214,2 KB
238 KB
261,8 KB
285,6 KB
309,4 KB
333,2 KB
357 KB
380,79 KB
404,59 KB
428,39 KB
452,19 KB
475,99 KB
499,79 KB
523,59 KB
547,39 KB
571,19 KB
596,35 KB
596,35 KB of 596,35 KB

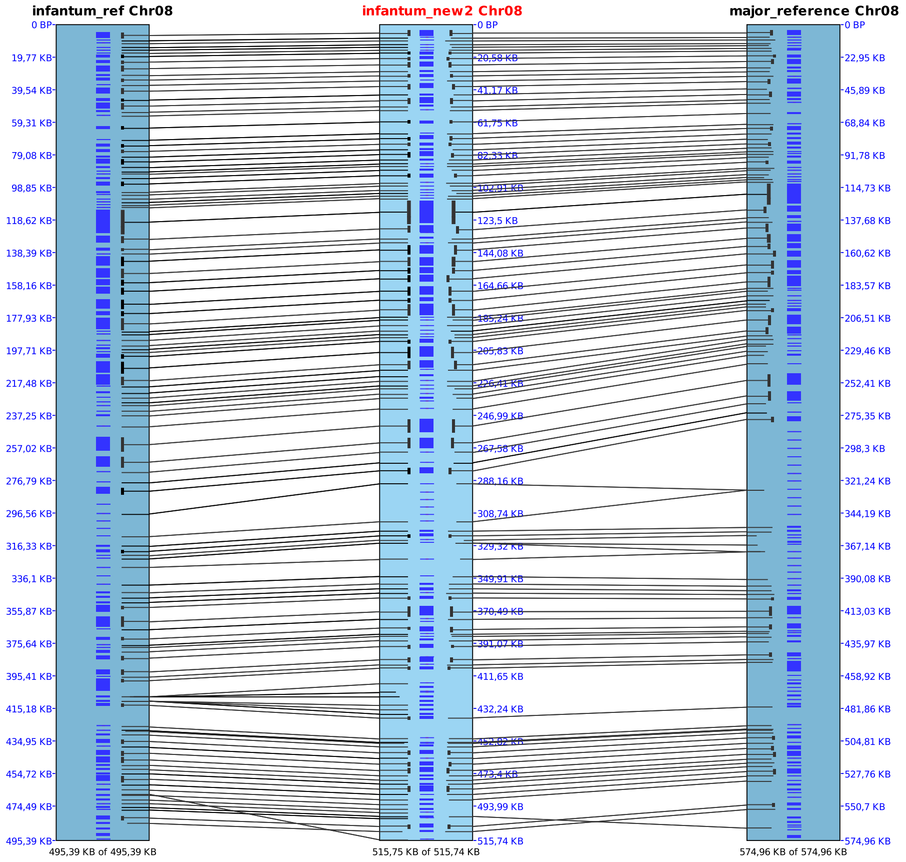
infantum_ref Chr08
infantum_new2 Chr08
major_reference Chr08
0 BP
19,77 KB
39,54 KB
59,31 KB
79,08 KB
98,85 KB
118,62 KB
138,39 KB
158,16 KB
177,93 KB
197,71 KB
217,48 KB
237,25 KB
257,02 KB
276,79 KB
296,56 KB
316,33 KB
336,1 KB
355,87 KB
375,64 KB
395,41 KB
415,18 KB
434,95 KB
454,72 KB
474,49 KB
495,39 KB
495,39 KB of 495,39 KB
0 BP
20,58 KB
41,17 KB
61,75 KB
82,33 KB
102,91 KB
123,5 KB
144,08 KB
164,66 KB
185,24 KB
205,83 KB
226,41 KB
246,99 KB
267,58 KB
288,16 KB
308,74 KB
329,32 KB
349,91 KB
370,49 KB
391,07 KB
411,65 KB
432,24 KB
452,82 KB
473,4 KB
493,99 KB
515,74 KB
515,75 KB of 515,74 KB
0 BP
22,95 KB
45,89 KB
68,84 KB
91,78 KB
114,73 KB
137,68 KB
160,62 KB
183,57 KB
206,51 KB
229,46 KB
252,41 KB
275,35 KB
298,3 KB
321,24 KB
344,19 KB
367,14 KB
390,08 KB
413,03 KB
435,97 KB
458,92 KB
481,86 KB
504,81 KB
527,76 KB
550,7 KB
574,96 KB
574,96 KB of 574,96 KB

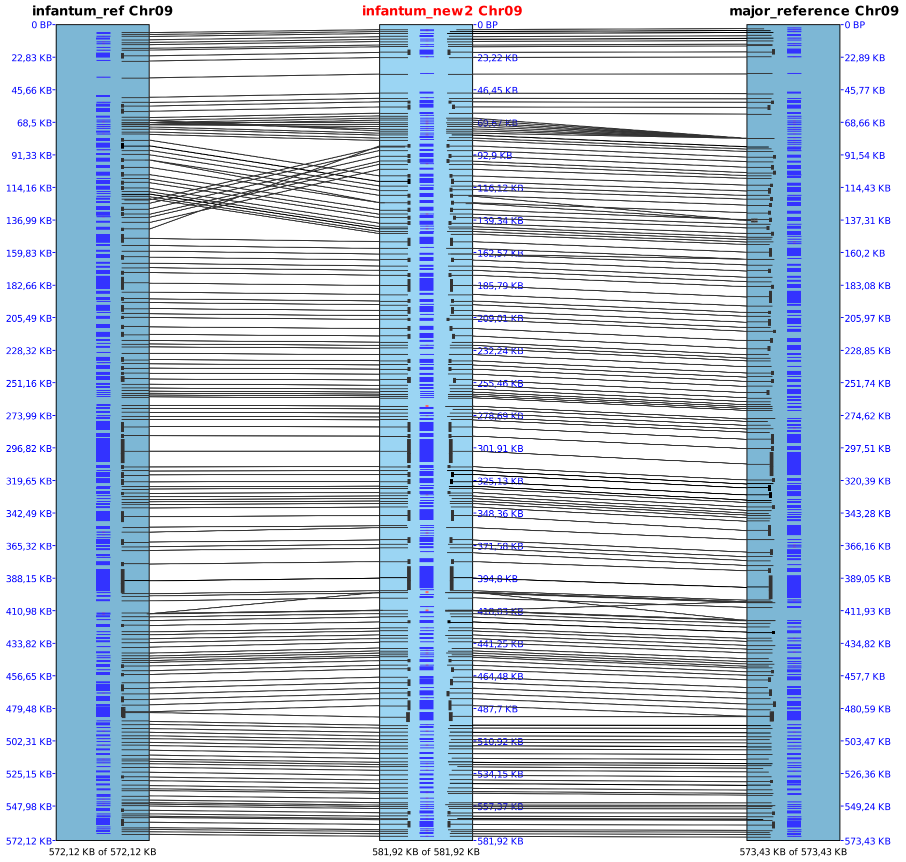

infantum_ref Chr09
infantum_new2 Chr09
major_reference Chr09
0 BP
22,83 KB
45,66 KB
68,5 KB
91,33 KB
114,16 KB
136,99 KB
159,83 KB
182,66 KB
205,49 KB
228,32 KB
251,16 KB
273,99 KB
296,82 KB
319,65 KB
342,49 KB
365,32 KB
388,15 KB
410,98 KB
433,82 KB
456,65 KB
479,48 KB
502,31 KB
525,15 KB
547,98 KB
572,12 KB
0 BP
23,22 KB
46,45 KB
69,67 KB
92,9 KB
116,12 KB
139,34 KB
162,57 KB
185,79 KB
209,01 KB
232,24 KB
255,46 KB
278,69 KB
301,91 KB
325,13 KB
348,36 KB
371,58 KB
394,8 KB
418,03 KB
441,25 KB
464,48 KB
487,7 KB
510,92 KB
534,15 KB
557,37 KB
581,92 KB
0 BP
22,89 KB
45,77 KB
68,66 KB
91,54 KB
114,43 KB
137,31 KB
160,2 KB
183,08 KB
205,97 KB
228,85 KB
251,74 KB
274,62 KB
297,51 KB
320,39 KB
343,28 KB
366,16 KB
389,05 KB
411,93 KB
434,82 KB
457,7 KB
480,59 KB
503,47 KB
526,36 KB
549,24 KB
573,43 KB
572,12 KB of 572,12 KB
581,92 KB of 581,92 KB
573,43 KB of 573,43 KB

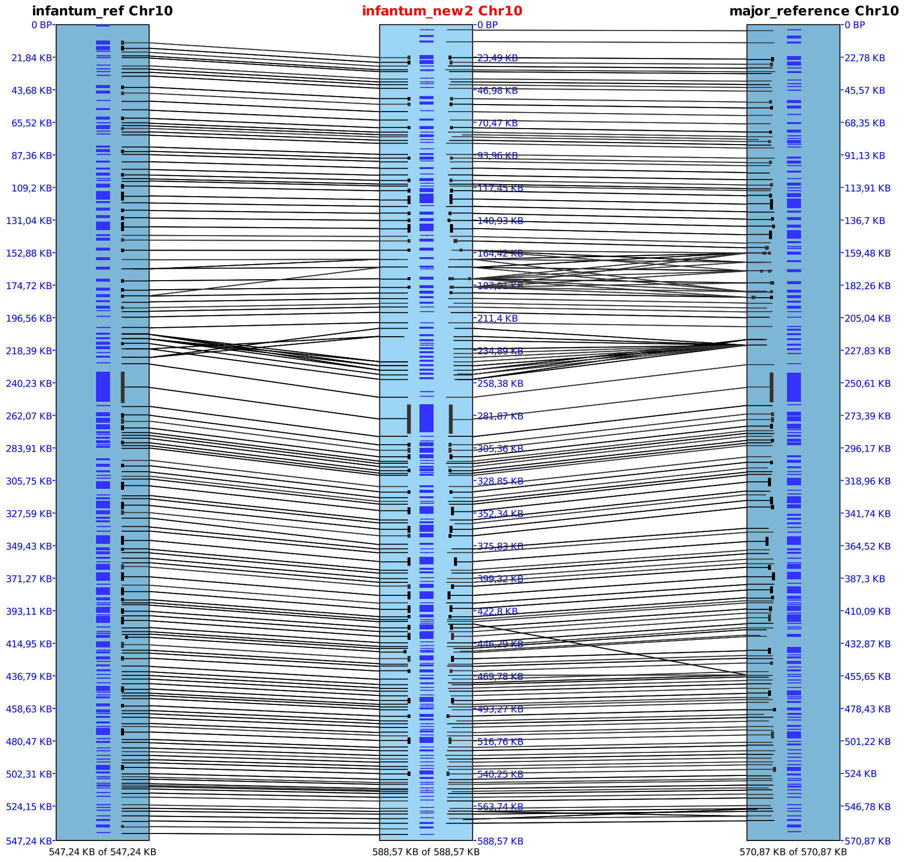

infantum_ref Chr10
infantum_new2 Chr10
major_reference Chr10
0 BP
21,84 KB
43,68 KB
65,52 KB
87,36 KB
109,2 KB
131,04 KB
152,88 KB
174,72 KB
196,56 KB
218,39 KB
240,23 KB
262,07 KB
283,91 KB
305,75 KB
327,59 KB
349,43 KB
371,27 KB
393,11 KB
414,95 KB
436,79 KB
458,63 KB
480,47 KB
502,31 KB
524,15 KB
547,24 KB
547,24 KB of 547,24 KB
0 BP
23,49 KB
46,98 KB
70,47 KB
93,96 KB
117,45 KB
140,93 KB
164,42 KB
187,91 KB
211,4 KB
234,89 KB
258,38 KB
281,87 KB
305,36 KB
328,85 KB
352,34 KB
375,83 KB
399,32 KB
422,8 KB
446,29 KB
469,78 KB
493,27 KB
516,76 KB
540,25 KB
563,74 KB
588,57 KB
588,57 KB of 588,57 KB
0 BP
22,78 KB
45,57 KB
68,35 KB
91,13 KB
113,91 KB
136,7 KB
159,48 KB
182,26 KB
205,04 KB
227,83 KB
250,61 KB
273,39 KB
296,17 KB
318,96 KB
341,74 KB
364,52 KB
387,3 KB
410,09 KB
432,87 KB
455,65 KB
478,43 KB
501,22 KB
524 KB
546,78 KB
570,87 KB
570,87 KB of 570,87 KB

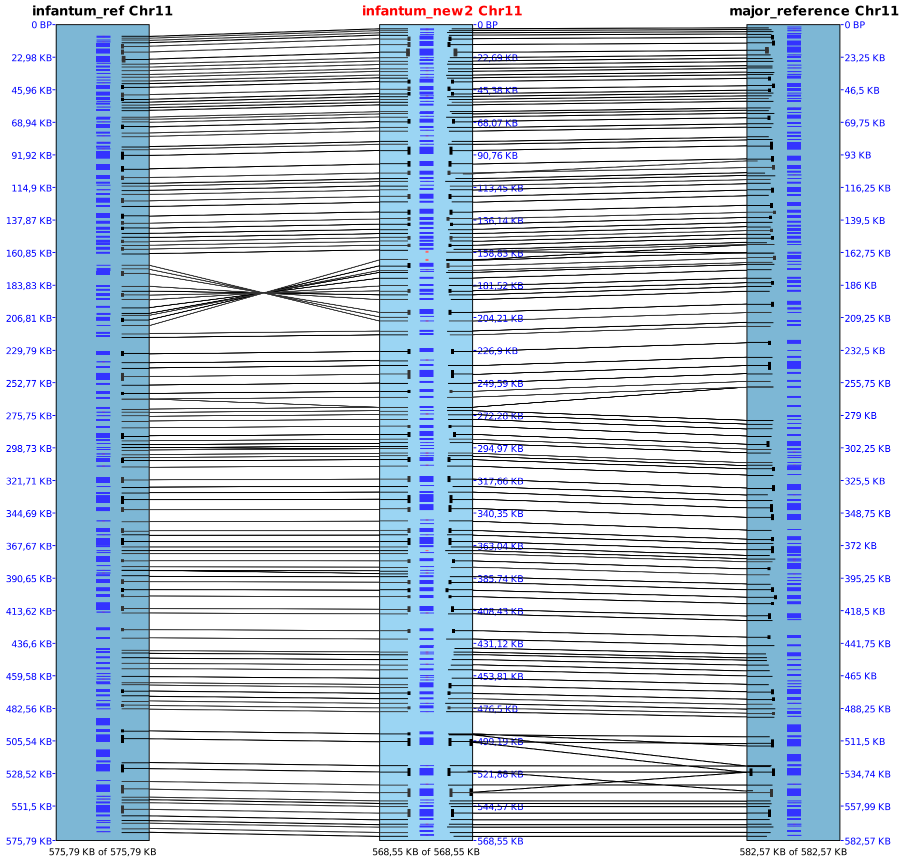
infantum_ref Chr11
infantum_new2 Chr11
major_reference Chr11
0 BP
22,98 KB
45,96 KB
68,94 KB
91,92 KB
114,9 KB
137,87 KB
160,85 KB
183,83 KB
206,81 KB
229,79 KB
252,77 KB
275,75 KB
298,73 KB
321,71 KB
344,69 KB
367,67 KB
390,65 KB
413,62 KB
436,6 KB
459,58 KB
482,56 KB
505,54 KB
528,52 KB
551,5 KB
575,79 KB
575,79 KB of 575,79 KB
0 BP
22,69 KB
45,38 KB
68,07 KB
90,76 KB
113,45 KB
136,14 KB
158,83 KB
181,52 KB
204,21 KB
226,9 KB
249,59 KB
272,28 KB
294,97 KB
317,66 KB
340,35 KB
363,04 KB
385,74 KB
408,43 KB
431,12 KB
453,81 KB
476,5 KB
499,19 KB
521,88 KB
544,57 KB
568,55 KB
568,55 KB of 568,55 KB
0 BP
23,25 KB
46,5 KB
69,75 KB
93 KB
116,25 KB
139,5 KB
162,75 KB
186 KB
209,25 KB
232,5 KB
255,75 KB
279 KB
302,25 KB
325,5 KB
348,75 KB
372 KB
395,25 KB
418,5 KB
441,75 KB
465 KB
488,25 KB
511,5 KB
534,74 KB
557,99 KB
582,57 KB
582,57 KB of 582,57 KB

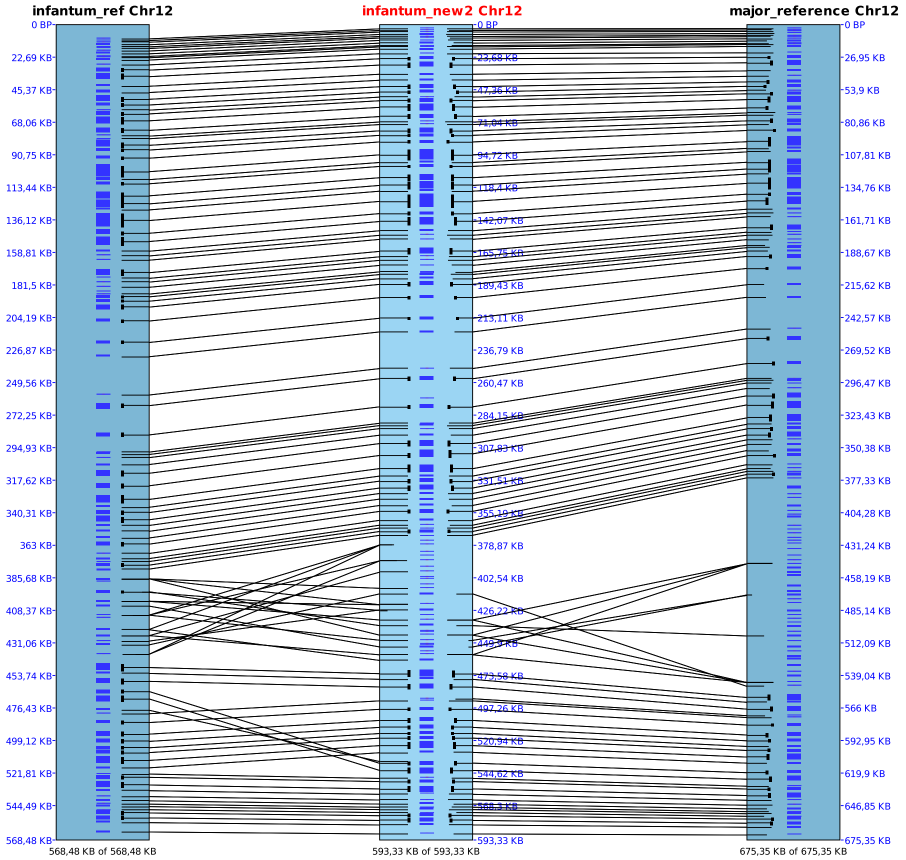

infantum_ref Chr12
infantum_new2 Chr12
major_reference Chr12
0 BP
22,69 KB
45,37 KB
68,06 KB
90,75 KB
113,44 KB
136,12 KB
158,81 KB
181,5 KB
204,19 KB
226,87 KB
249,56 KB
272,25 KB
294,93 KB
317,62 KB
340,31 KB
363 KB
385,68 KB
408,37 KB
431,06 KB
453,74 KB
476,43 KB
499,12 KB
521,81 KB
544,49 KB
568,48 KB
568,48 KB of 568,48 KB
0 BP
23,68 KB
47,36 KB
71,04 KB
94,72 KB
118,4 KB
142,07 KB
165,75 KB
189,43 KB
213,11 KB
236,79 KB
260,47 KB
284,15 KB
307,83 KB
331,51 KB
355,19 KB
378,87 KB
402,54 KB
426,22 KB
449,9 KB
473,58 KB
497,26 KB
520,94 KB
544,62 KB
568,3 KB
593,33 KB
593,33 KB of 593,33 KB
0 BP
26,95 KB
53,9 KB
80,86 KB
107,81 KB
134,76 KB
161,71 KB
188,67 KB
215,62 KB
242,57 KB
269,52 KB
296,47 KB
323,43 KB
350,38 KB
377,33 KB
404,28 KB
431,24 KB
458,19 KB
485,14 KB
512,09 KB
539,04 KB
566 KB
592,95 KB
619,9 KB
646,85 KB
675,35 KB
675,35 KB of 675,35 KB

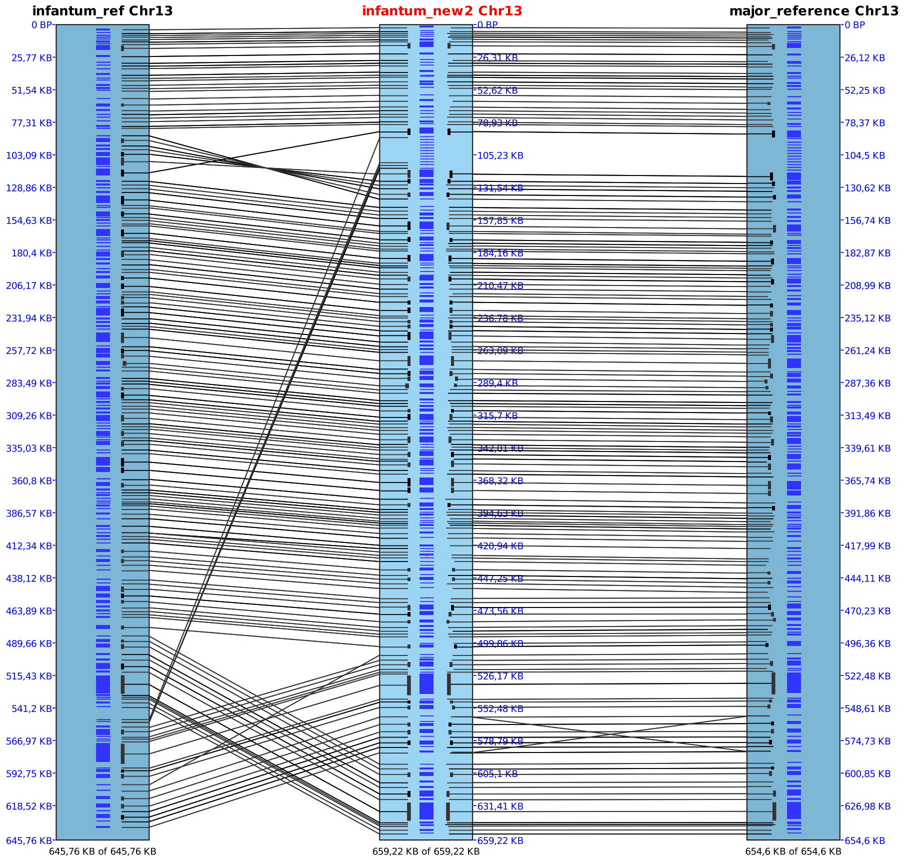
infantum_ref Chr13
infantum_new2 Chr13
major_reference Chr13
0 BP
25,77 KB
51,54 KB
77,31 KB
103,09 KB
128,86 KB
154,63 KB
180,4 KB
206,17 KB
231,94 KB
257,72 KB
283,49 KB
309,26 KB
335,03 KB
360,8 KB
386,57 KB
412,34 KB
438,12 KB
463,89 KB
489,66 KB
515,43 KB
541,2 KB
566,97 KB
592,75 KB
618,52 KB
645,76 KB
645,76 KB of 645,76 KB
0 BP
26,31 KB
52,62 KB
78,93 KB
105,23 KB
131,54 KB
157,85 KB
184,16 KB
210,47 KB
236,78 KB
263,09 KB
289,4 KB
315,7 KB
342,01 KB
368,32 KB
394,63 KB
420,94 KB
447,25 KB
473,56 KB
499,86 KB
526,17 KB
552,48 KB
578,79 KB
605,1 KB
631,41 KB
659,22 KB
659,22 KB of 659,22 KB
0 BP
26,12 KB
52,25 KB
78,37 KB
104,5 KB
130,62 KB
156,74 KB
182,87 KB
208,99 KB
235,12 KB
261,24 KB
287,36 KB
313,49 KB
339,61 KB
365,74 KB
391,86 KB
417,99 KB
444,11 KB
470,23 KB
496,36 KB
522,48 KB
548,61 KB
574,73 KB
600,85 KB
626,98 KB
654,6 KB
654,6 KB of 654,6 KB

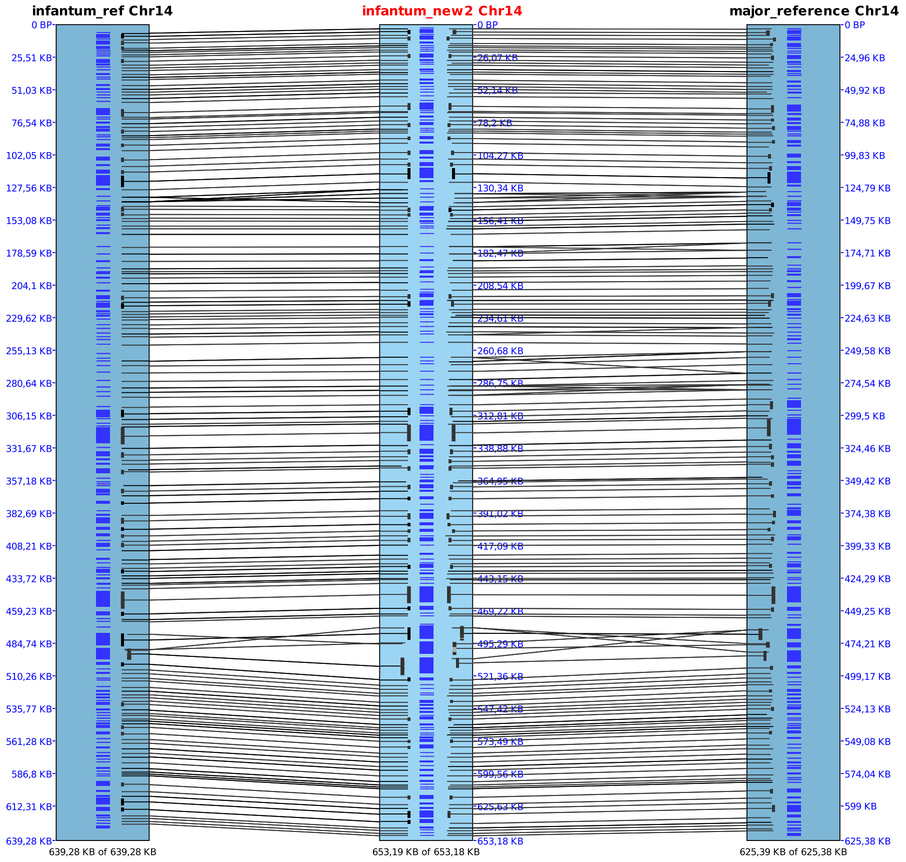
infantum_ref Chr14
infantum_new2 Chr14
major_reference Chr14
0 BP
25,51 KB
51,03 KB
76,54 KB
102,05 KB
127,56 KB
153,08 KB
178,59 KB
204,1 KB
229,62 KB
255,13 KB
280,64 KB
306,15 KB
331,67 KB
357,18 KB
382,69 KB
408,21 KB
433,72 KB
459,23 KB
484,74 KB
510,26 KB
535,77 KB
561,28 KB
586,8 KB
612,31 KB
639,28 KB
639,28 KB of 639,28 KB
0 BP
26,07 KB
52,14 KB
78,2 KB
104,27 KB
130,34 KB
156,41 KB
182,47 KB
208,54 KB
234,61 KB
260,68 KB
286,75 KB
312,81 KB
338,88 KB
364,95 KB
391,02 KB
417,09 KB
443,15 KB
469,22 KB
495,29 KB
521,36 KB
547,42 KB
573,49 KB
599,56 KB
625,63 KB
653,18 KB
653,19 KB of 653,18 KB
0 BP
24,96 KB
49,92 KB
74,88 KB
99,83 KB
124,79 KB
149,75 KB
174,71 KB
199,67 KB
224,63 KB
249,58 KB
274,54 KB
299,5 KB
324,46 KB
349,42 KB
374,38 KB
399,33 KB
424,29 KB
449,25 KB
474,21 KB
499,17 KB
524,13 KB
549,08 KB
574,04 KB
599 KB
625,38 KB
625,39 KB of 625,38 KB

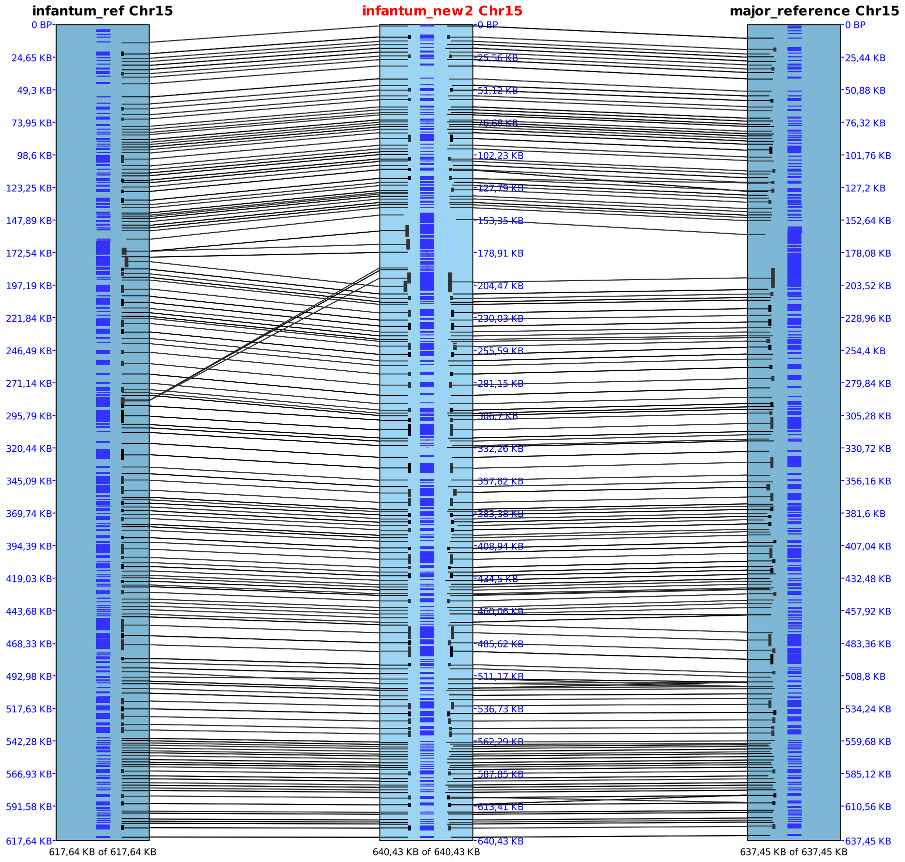

infantum_ref Chr15
infantum_new2 Chr15
major_reference Chr15
0 BP
24,65 KB
49,3 KB
73,95 KB
98,6 KB
123,25 KB
147,89 KB
172,54 KB
197,19 KB
221,84 KB
246,49 KB
271,14 KB
295,79 KB
320,44 KB
345,09 KB
369,74 KB
394,39 KB
419,03 KB
443,68 KB
468,33 KB
492,98 KB
517,63 KB
542,28 KB
566,93 KB
591,58 KB
617,64 KB
617,64 KB of 617,64 KB
0 BP
25,56 KB
51,12 KB
76,68 KB
102,23 KB
127,79 KB
153,35 KB
178,91 KB
204,47 KB
230,03 KB
255,59 KB
281,15 KB
306,7 KB
332,26 KB
357,82 KB
383,38 KB
408,94 KB
434,5 KB
460,06 KB
485,62 KB
511,17 KB
536,73 KB
562,29 KB
587,85 KB
613,41 KB
640,43 KB
640,43 KB of 640,43 KB
0 BP
25,44 KB
50,88 KB
76,32 KB
101,76 KB
127,2 KB
152,64 KB
178,08 KB
203,52 KB
228,96 KB
254,4 KB
279,84 KB
305,28 KB
330,72 KB
356,16 KB
381,6 KB
407,04 KB
432,48 KB
457,92 KB
483,36 KB
508,8 KB
534,24 KB
559,68 KB
585,12 KB
610,56 KB
637,45 KB
637,45 KB of 637,45 KB

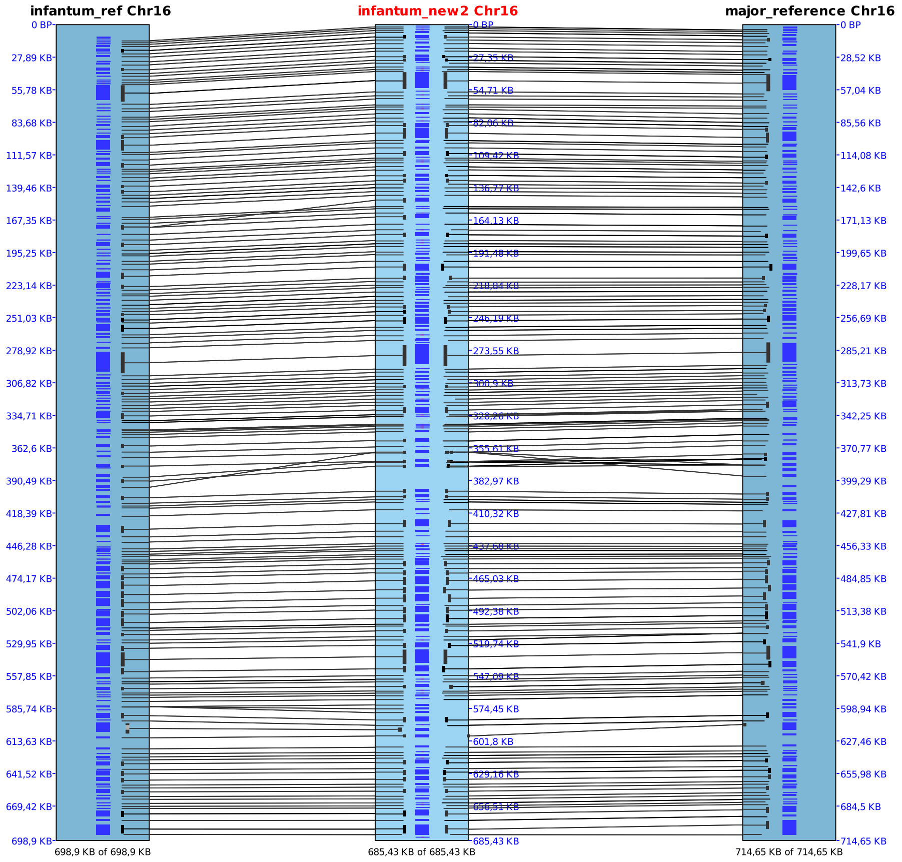

infantum_ref Chr16
infantum_new2 Chr16
major_reference Chr16
0 BP
27,89 KB
55,78 KB
83,68 KB
111,57 KB
139,46 KB
167,35 KB
195,25 KB
223,14 KB
251,03 KB
278,92 KB
306,82 KB
334,71 KB
362,6 KB
390,49 KB
418,39 KB
446,28 KB
474,17 KB
502,06 KB
529,95 KB
557,85 KB
585,74 KB
613,63 KB
641,52 KB
669,42 KB
698,9 KB
0 BP
27,35 KB
54,71 KB
82,06 KB
109,42 KB
136,77 KB
164,13 KB
191,48 KB
218,84 KB
246,19 KB
273,55 KB
300,9 KB
328,26 KB
355,61 KB
382,97 KB
410,32 KB
437,68 KB
465,03 KB
492,38 KB
519,74 KB
547,09 KB
574,45 KB
601,8 KB
629,16 KB
656,51 KB
685,43 KB
0 BP
28,52 KB
57,04 KB
85,56 KB
114,08 KB
142,6 KB
171,13 KB
199,65 KB
228,17 KB
256,69 KB
285,21 KB
313,73 KB
342,25 KB
370,77 KB
399,29 KB
427,81 KB
456,33 KB
484,85 KB
513,38 KB
541,9 KB
570,42 KB
598,94 KB
627,46 KB
655,98 KB
684,5 KB
714,65 KB
698,9 KB of 698,9 KB
685,43 KB of 685,43 KB
714,65 KB of 714,65 KB

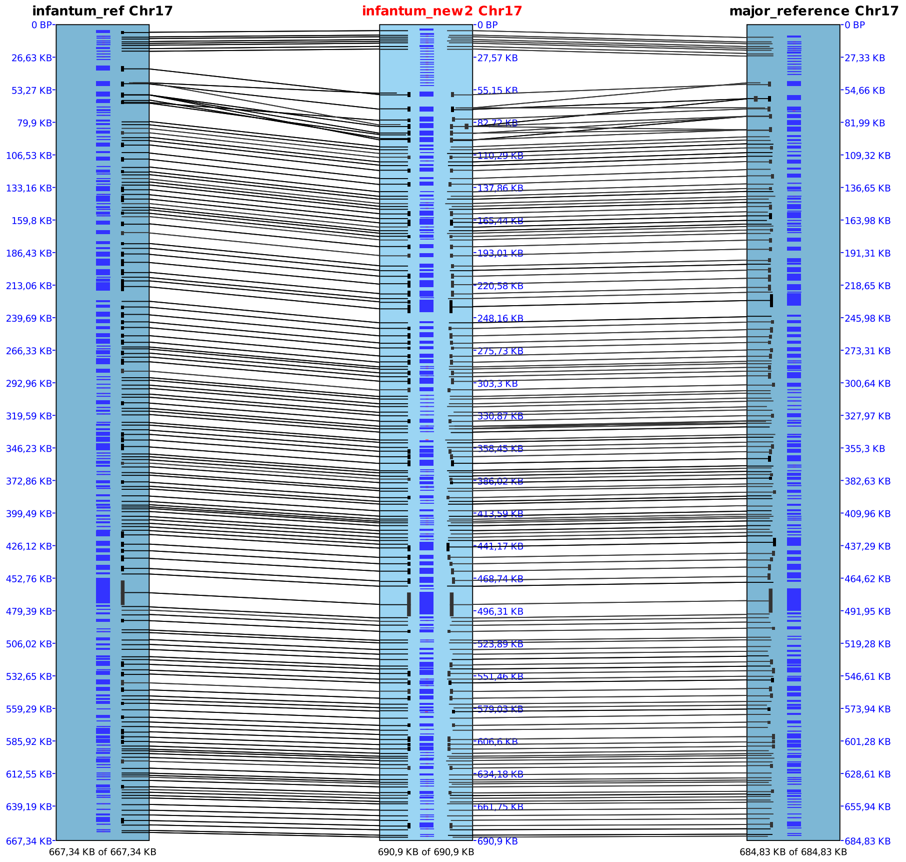
infantum_ref Chr17
infantum_new2 Chr17
major_reference Chr17
0 BP
26,63 KB
53,27 KB
79,9 KB
106,53 KB
133,16 KB
159,8 KB
186,43 KB
213,06 KB
239,69 KB
266,33 KB
292,96 KB
319,59 KB
346,23 KB
372,86 KB
399,49 KB
426,12 KB
452,76 KB
479,39 KB
506,02 KB
532,65 KB
559,29 KB
585,92 KB
612,55 KB
639,19 KB
667,34 KB
0 BP
27,57 KB
55,15 KB
82,72 KB
110,29 KB
137,86 KB
165,44 KB
193,01 KB
220,58 KB
248,16 KB
275,73 KB
303,3 KB
330,87 KB
358,45 KB
386,02 KB
413,59 KB
441,17 KB
468,74 KB
496,31 KB
523,89 KB
551,46 KB
579,03 KB
606,6 KB
634,18 KB
661,75 KB
690,9 KB
0 BP
27,33 KB
54,66 KB
81,99 KB
109,32 KB
136,65 KB
163,98 KB
191,31 KB
218,65 KB
245,98 KB
273,31 KB
300,64 KB
327,97 KB
355,3 KB
382,63 KB
409,96 KB
437,29 KB
464,62 KB
491,95 KB
519,28 KB
546,61 KB
573,94 KB
601,28 KB
628,61 KB
655,94 KB
684,83 KB
667,34 KB of 667,34 KB
690,9 KB of 690,9 KB
684,83 KB of 684,83 KB

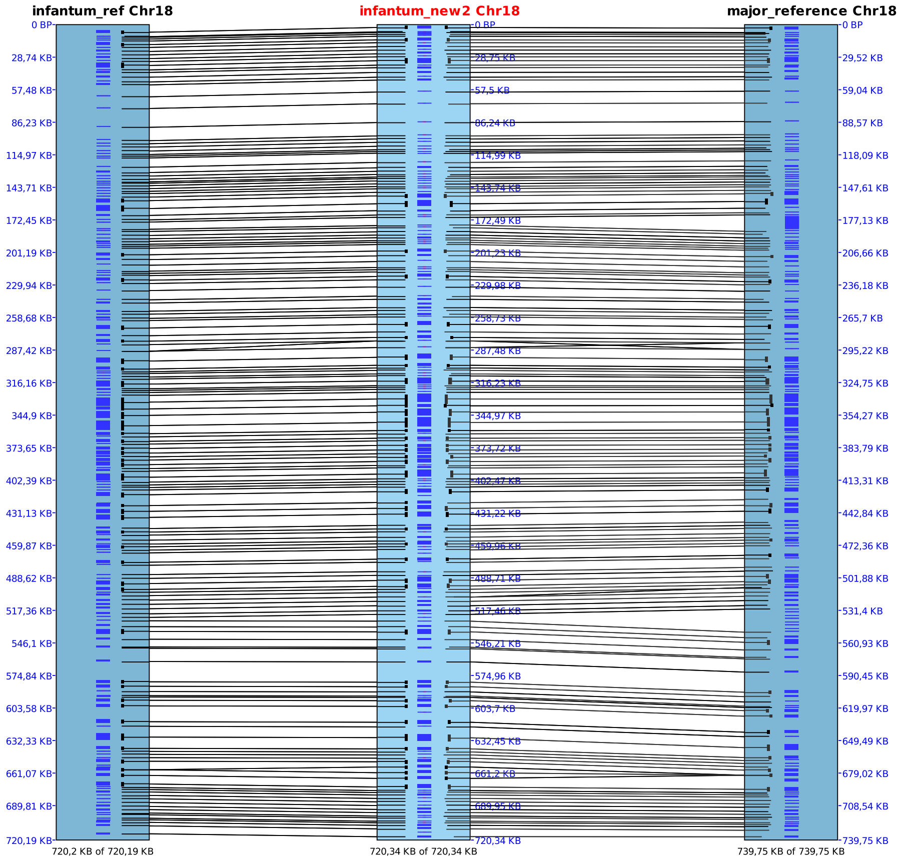
infantum_ref Chr18
infantum_new2 Chr18
major_reference Chr18
0 BP
28,74 KB
57,48 KB
86,23 KB
114,97 KB
143,71 KB
172,45 KB
201,19 KB
229,94 KB
258,68 KB
287,42 KB
316,16 KB
344,9 KB
373,65 KB
402,39 KB
431,13 KB
459,87 KB
488,62 KB
517,36 KB
546,1 KB
574,84 KB
603,58 KB
632,33 KB
661,07 KB
689,81 KB
720,19 KB
720,2 KB of 720,19 KB
0 BP
28,75 KB
57,5 KB
86,24 KB
114,99 KB
143,74 KB
172,49 KB
201,23 KB
229,98 KB
258,73 KB
287,48 KB
316,23 KB
344,97 KB
373,72 KB
402,47 KB
431,22 KB
459,96 KB
488,71 KB
517,46 KB
546,21 KB
574,96 KB
603,7 KB
632,45 KB
661,2 KB
689,95 KB
720,34 KB
720,34 KB of 720,34 KB
0 BP
29,52 KB
59,04 KB
88,57 KB
118,09 KB
147,61 KB
177,13 KB
206,66 KB
236,18 KB
265,7 KB
295,22 KB
324,75 KB
354,27 KB
383,79 KB
413,31 KB
442,84 KB
472,36 KB
501,88 KB
531,4 KB
560,93 KB
590,45 KB
619,97 KB
649,49 KB
679,02 KB
708,54 KB
739,75 KB
739,75 KB of 739,75 KB

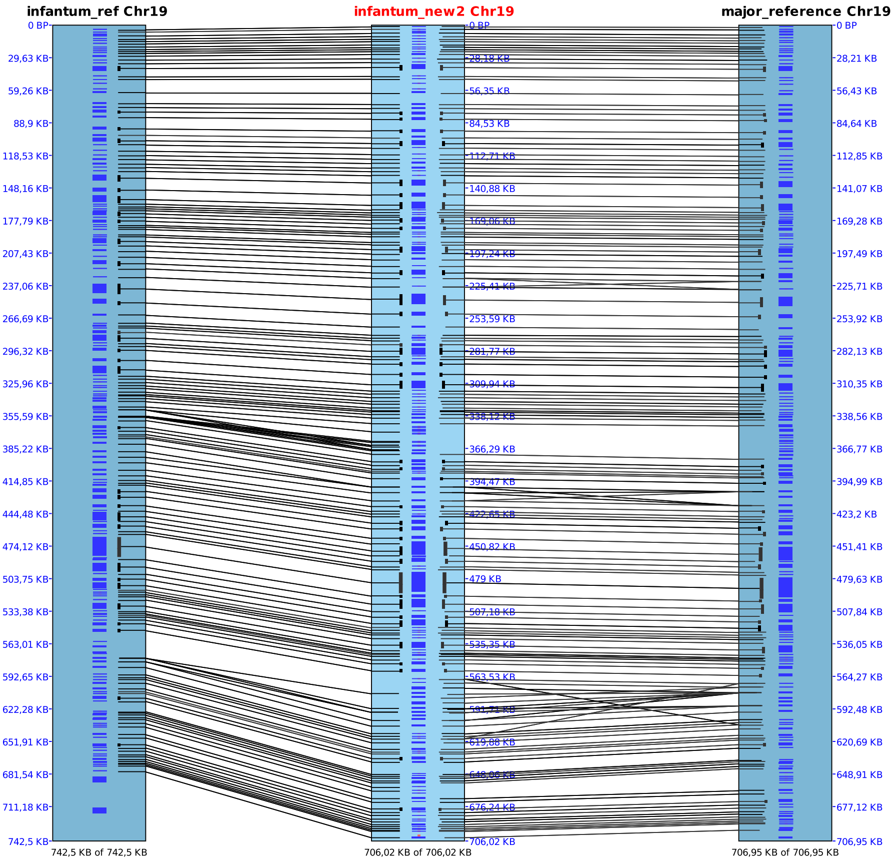
infantum_ref Chr19
infantum_new2 Chr19
major_reference Chr19
0 BP
29,63 KB
59,26 KB
88,9 KB
118,53 KB
148,16 KB
177,79 KB
207,43 KB
237,06 KB
266,69 KB
296,32 KB
325,96 KB
355,59 KB
385,22 KB
414,85 KB
444,48 KB
474,12 KB
503,75 KB
533,38 KB
563,01 KB
592,65 KB
622,28 KB
651,91 KB
681,54 KB
711,18 KB
742,5 KB
742,5 KB of 742,5 KB
0 BP
28,18 KB
56,35 KB
84,53 KB
112,71 KB
140,88 KB
169,06 KB
197,24 KB
225,41 KB
253,59 KB
281,77 KB
309,94 KB
338,12 KB
366,29 KB
394,47 KB
422,65 KB
450,82 KB
479 KB
507,18 KB
535,35 KB
563,53 KB
591,71 KB
619,88 KB
648,06 KB
676,24 KB
706,02 KB
706,02 KB of 706,02 KB
0 BP
28,21 KB
56,43 KB
84,64 KB
112,85 KB
141,07 KB
169,28 KB
197,49 KB
225,71 KB
253,92 KB
282,13 KB
310,35 KB
338,56 KB
366,77 KB
394,99 KB
423,2 KB
451,41 KB
479,63 KB
507,84 KB
536,05 KB
564,27 KB
592,48 KB
620,69 KB
648,91 KB
677,12 KB
706,95 KB
706,95 KB of 706,95 KB

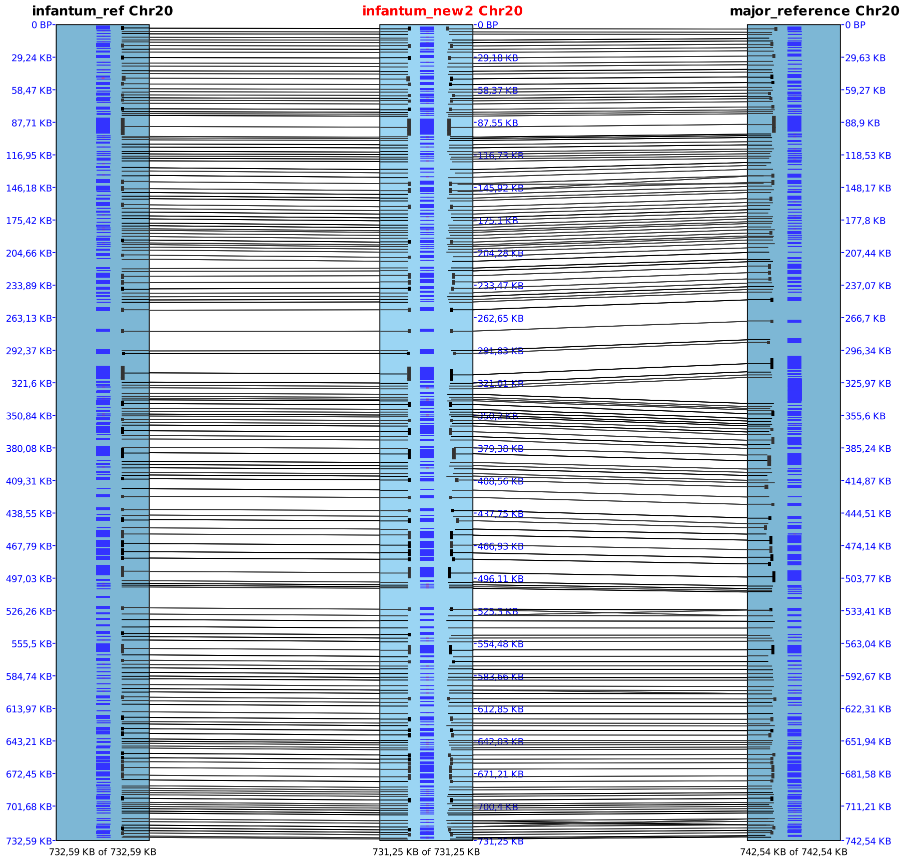

infantum_ref Chr20
infantum_new2 Chr20
major_reference Chr20
0 BP
29,24 KB
58,47 KB
87,71 KB
116,95 KB
146,18 KB
175,42 KB
204,66 KB
233,89 KB
263,13 KB
292,37 KB
321,6 KB
350,84 KB
380,08 KB
409,31 KB
438,55 KB
467,79 KB
497,03 KB
526,26 KB
555,5 KB
584,74 KB
613,97 KB
643,21 KB
672,45 KB
701,68 KB
732,59 KB
0 BP
29,18 KB
58,37 KB
87,55 KB
116,73 KB
145,92 KB
175,1 KB
204,28 KB
233,47 KB
262,65 KB
291,83 KB
321,01 KB
350,2 KB
379,38 KB
408,56 KB
437,75 KB
466,93 KB
496,11 KB
525,3 KB
554,48 KB
583,66 KB
612,85 KB
642,03 KB
671,21 KB
700,4 KB
731,25 KB
0 BP
29,63 KB
59,27 KB
88,9 KB
118,53 KB
148,17 KB
177,8 KB
207,44 KB
237,07 KB
266,7 KB
296,34 KB
325,97 KB
355,6 KB
385,24 KB
414,87 KB
444,51 KB
474,14 KB
503,77 KB
533,41 KB
563,04 KB
592,67 KB
622,31 KB
651,94 KB
681,58 KB
711,21 KB
742,54 KB
732,59 KB of 732,59 KB
731,25 KB of 731,25 KB
742,54 KB of 742,54 KB

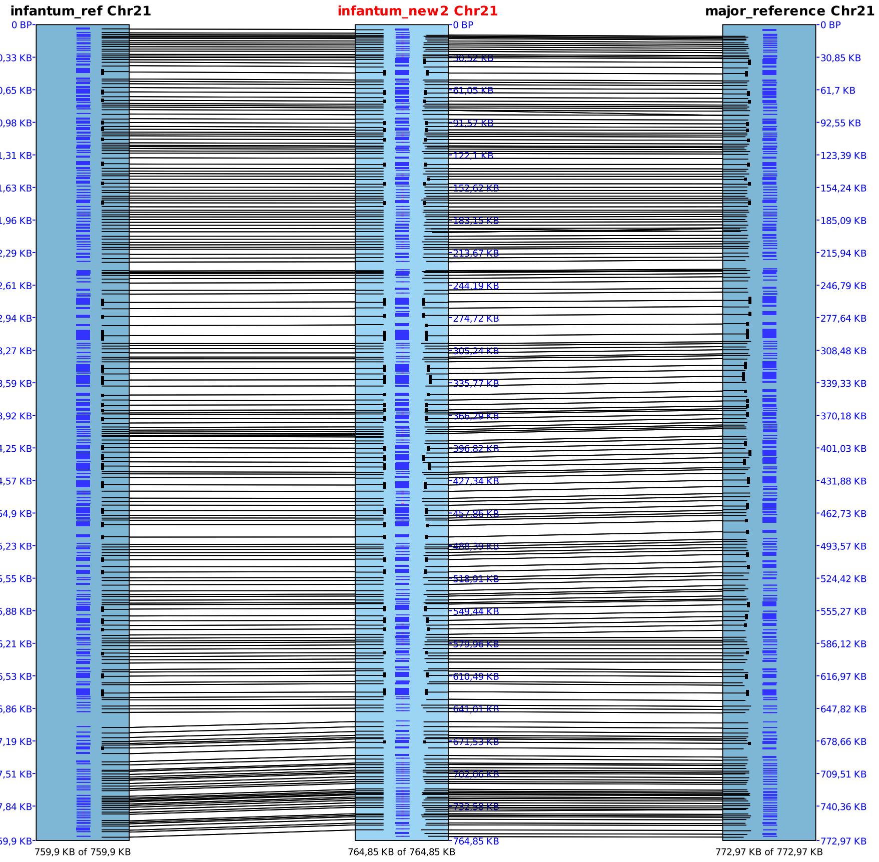

infantum_ref Chr21
infantum_new2 Chr21
major_reference Chr21
0 BP
30,33 KB
60,65 KB
90,98 KB
121,31 KB
151,63 KB
181,96 KB
212,29 KB
242,61 KB
272,94 KB
303,27 KB
333,59 KB
363,92 KB
394,25 KB
424,57 KB
454,9 KB
485,23 KB
515,55 KB
545,88 KB
576,21 KB
606,53 KB
636,86 KB
667,19 KB
697,51 KB
727,84 KB
759,9 KB
759,9 KB of 759,9 KB
0 BP
30,52 KB
61,05 KB
91,57 KB
122,1 KB
152,62 KB
183,15 KB
213,67 KB
244,19 KB
274,72 KB
305,24 KB
335,77 KB
366,29 KB
396,82 KB
427,34 KB
457,86 KB
488,39 KB
518,91 KB
549,44 KB
579,96 KB
610,49 KB
641,01 KB
671,53 KB
702,06 KB
732,58 KB
764,85 KB
764,85 KB of 764,85 KB
0 BP
30,85 KB
61,7 KB
92,55 KB
123,39 KB
154,24 KB
185,09 KB
215,94 KB
246,79 KB
277,64 KB
308,48 KB
339,33 KB
370,18 KB
401,03 KB
431,88 KB
462,73 KB
493,57 KB
524,42 KB
555,27 KB
586,12 KB
616,97 KB
647,82 KB
678,66 KB
709,51 KB
740,36 KB
772,97 KB
772,97 KB of 772,97 KB

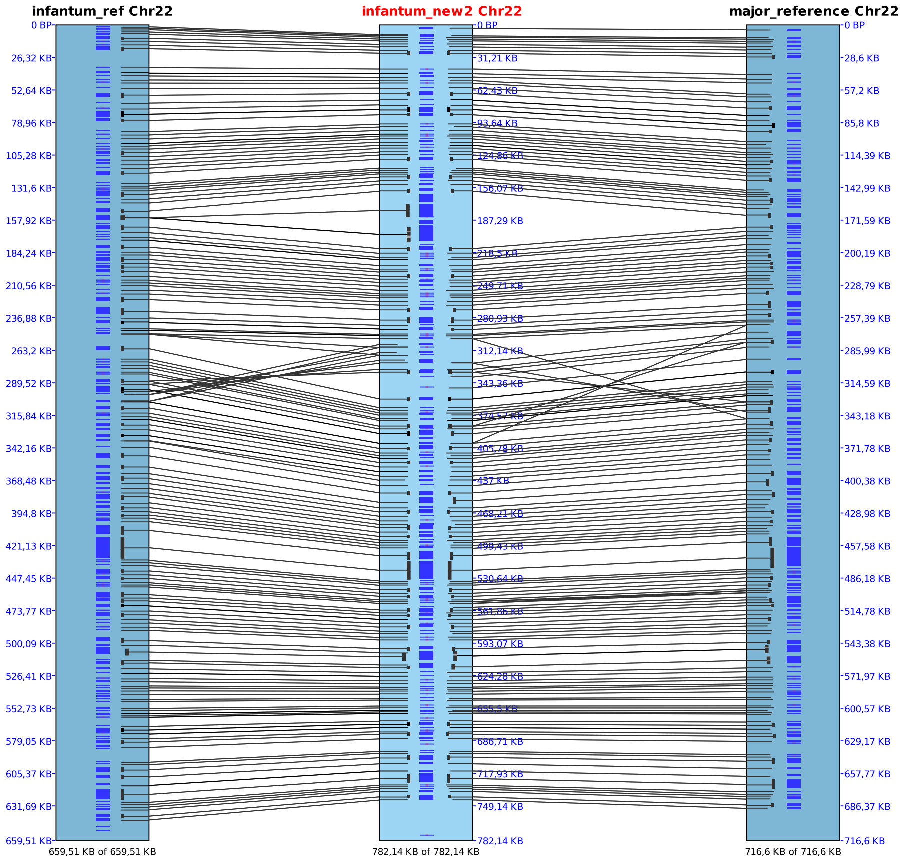

infantum_ref Chr22
infantum_new2 Chr22
major_reference Chr22
0 BP
26,32 KB
52,64 KB
78,96 KB
105,28 KB
131,6 KB
157,92 KB
184,24 KB
210,56 KB
236,88 KB
263,2 KB
289,52 KB
315,84 KB
342,16 KB
368,48 KB
394,8 KB
421,13 KB
447,45 KB
473,77 KB
500,09 KB
526,41 KB
552,73 KB
579,05 KB
605,37 KB
631,69 KB
659,51 KB
659,51 KB of 659,51 KB
0 BP
31,21 KB
62,43 KB
93,64 KB
124,86 KB
156,07 KB
187,29 KB
218,5 KB
249,71 KB
280,93 KB
312,14 KB
343,36 KB
374,57 KB
405,78 KB
437 KB
468,21 KB
499,43 KB
530,64 KB
561,86 KB
593,07 KB
624,28 KB
655,5 KB
686,71 KB
717,93 KB
749,14 KB
782,14 KB
782,14 KB of 782,14 KB
0 BP
28,6 KB
57,2 KB
85,8 KB
114,39 KB
142,99 KB
171,59 KB
200,19 KB
228,79 KB
257,39 KB
285,99 KB
314,59 KB
343,18 KB
371,78 KB
400,38 KB
428,98 KB
457,58 KB
486,18 KB
514,78 KB
543,38 KB
571,97 KB
600,57 KB
629,17 KB
657,77 KB
686,37 KB
716,6 KB
716,6 KB of 716,6 KB

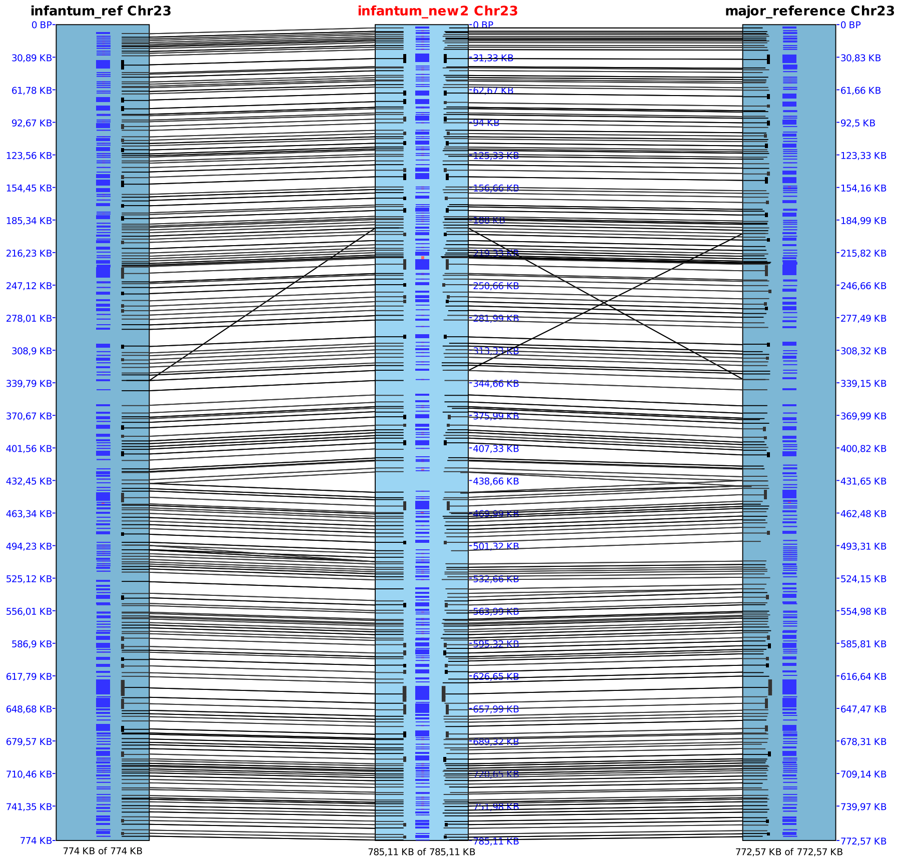

infantum_ref Chr23
infantum_new2 Chr23
major_reference Chr23
0 BP
30,89 KB
61,78 KB
92,67 KB
123,56 KB
154,45 KB
185,34 KB
216,23 KB
247,12 KB
278,01 KB
308,9 KB
339,79 KB
370,67 KB
401,56 KB
432,45 KB
463,34 KB
494,23 KB
525,12 KB
556,01 KB
586,9 KB
617,79 KB
648,68 KB
679,57 KB
710,46 KB
741,35 KB
774 KB
774 KB of 774 KB
0 BP
31,33 KB
62,67 KB
94 KB
125,33 KB
156,66 KB
188 KB
250,66 KB
281,99 KB
313,33 KB
344,66 KB
375,99 KB
407,33 KB
438,66 KB
501,32 KB
532,66 KB
563,99 KB
595,32 KB
626,65 KB
657,99 KB
689,32 KB
720,65 KB
751,98 KB
785,11 KB
785,11 KB of 785,11 KB
0 BP
30,83 KB
61,66 KB
92,5 KB
123,33 KB
154,16 KB
184,99 KB
215,82 KB
246,66 KB
277,49 KB
308,32 KB
339,15 KB
369,99 KB
400,82 KB
431,65 KB
462,48 KB
493,31 KB
524,15 KB
554,98 KB
585,81 KB
616,64 KB
647,47 KB
678,31 KB
709,14 KB
739,97 KB
772,57 KB
772,57 KB of 772,57 KB

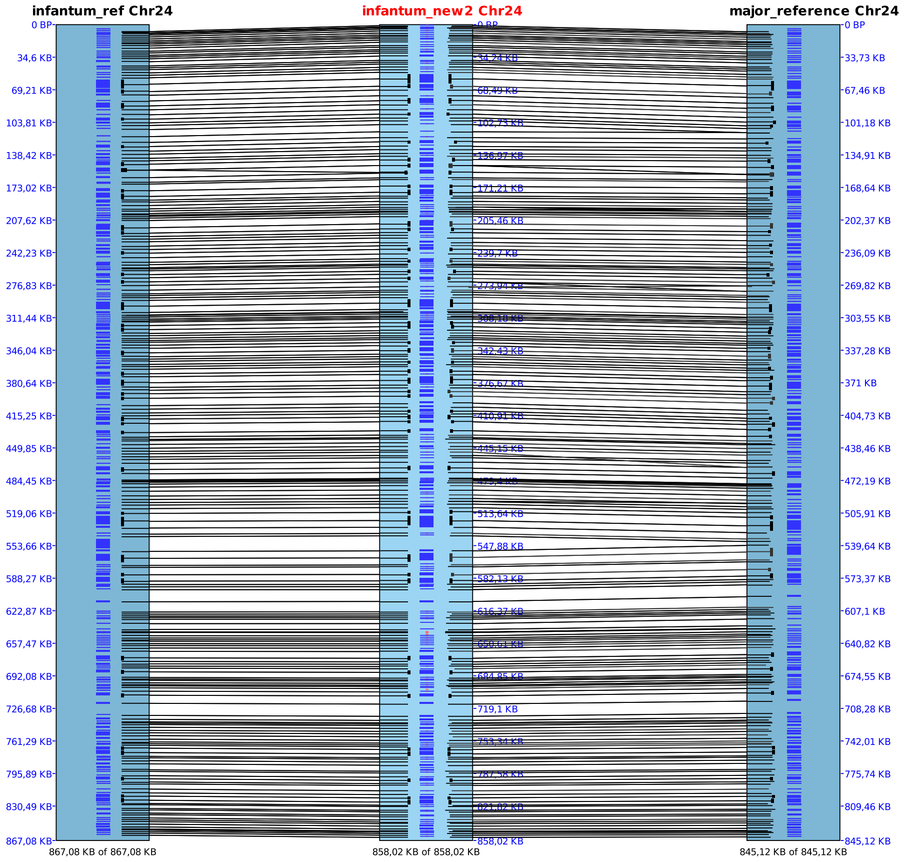
infantum_ref Chr24
infantum_new2 Chr24
major_reference Chr24
0 BP
34,6 KB
69,21 KB
103,81 KB
138,42 KB
173,02 KB
207,62 KB
242,23 KB
276,83 KB
311,44 KB
346,04 KB
380,64 KB
415,25 KB
449,85 KB
484,45 KB
519,06 KB
553,66 KB
588,27 KB
622,87 KB
657,47 KB
692,08 KB
726,68 KB
761,29 KB
795,89 KB
830,49 KB
867,08 KB
867,08 KB of 867,08 KB
0 BP
34,24 KB
68,49 KB
102,73 KB
136,97 KB
171,21 KB
205,46 KB
239,7 KB
273,94 KB
308,18 KB
342,43 KB
376,67 KB
410,91 KB
445,15 KB
479,4 KB
513,64 KB
547,88 KB
582,13 KB
616,37 KB
650,61 KB
684,85 KB
719,1 KB
753,34 KB
787,58 KB
821,82 KB
858,02 KB
858,02 KB of 858,02 KB
0 BP
33,73 KB
67,46 KB
101,18 KB
134,91 KB
168,64 KB
202,37 KB
236,09 KB
269,82 KB
303,55 KB
337,28 KB
371 KB
404,73 KB
438,46 KB
472,19 KB
505,91 KB
539,64 KB
573,37 KB
607,1 KB
640,82 KB
674,55 KB
708,28 KB
742,01 KB
775,74 KB
809,46 KB
845,12 KB
845,12 KB of 845,12 KB

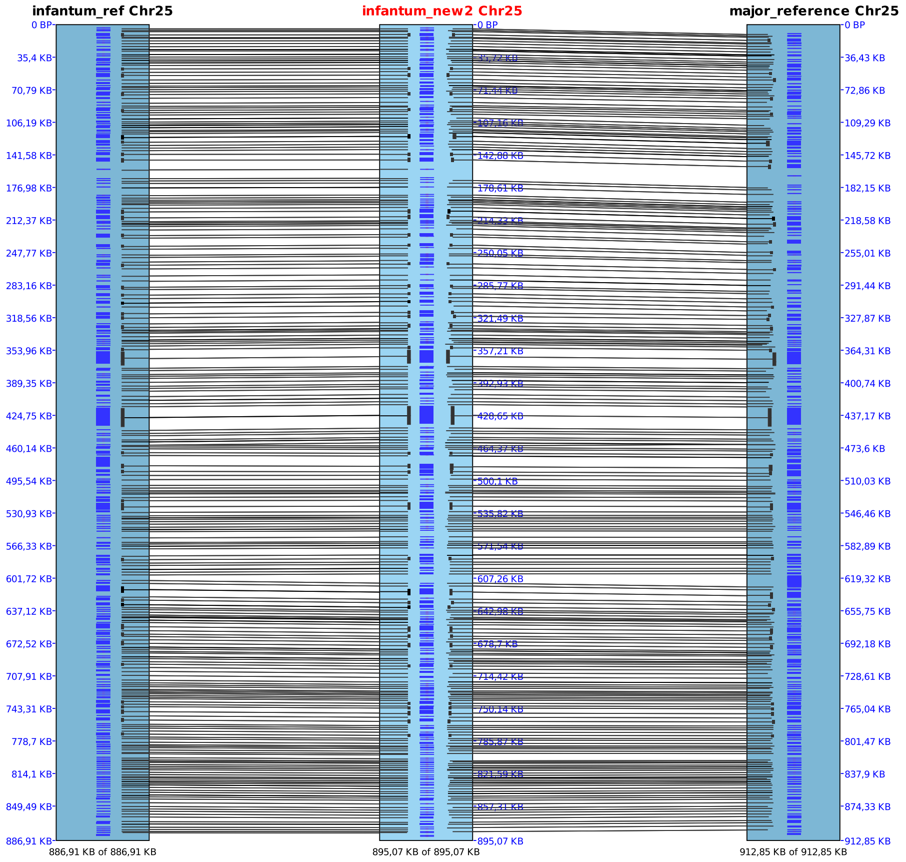

infantum_ref Chr25
infantum_new2 Chr25
major_reference Chr25
0 BP
35,4 KB
70,79 KB
106,19 KB
141,58 KB
176,98 KB
212,37 KB
247,77 KB
283,16 KB
318,56 KB
353,96 KB
389,35 KB
424,75 KB
460,14 KB
495,54 KB
530,93 KB
566,33 KB
601,72 KB
637,12 KB
672,52 KB
707,91 KB
743,31 KB
778,7 KB
814,1 KB
849,49 KB
886,91 KB
886,91 KB of 886,91 KB
0 BP
35,72 KB
71,44 KB
107,16 KB
142,88 KB
178,61 KB
214,33 KB
250,05 KB
285,77 KB
321,49 KB
357,21 KB
392,93 KB
428,65 KB
464,37 KB
500,1 KB
535,82 KB
571,54 KB
607,26 KB
642,98 KB
678,7 KB
714,42 KB
750,14 KB
785,87 KB
821,59 KB
857,31 KB
895,07 KB
895,07 KB of 895,07 KB
0 BP
36,43 KB
72,86 KB
109,29 KB
145,72 KB
182,15 KB
218,58 KB
255,01 KB
291,44 KB
327,87 KB
364,31 KB
400,74 KB
437,17 KB
473,6 KB
510,03 KB
546,46 KB
582,89 KB
619,32 KB
655,75 KB
692,18 KB
728,61 KB
765,04 KB
801,47 KB
837,9 KB
874,33 KB
912,85 KB
912,85 KB of 912,85 KB

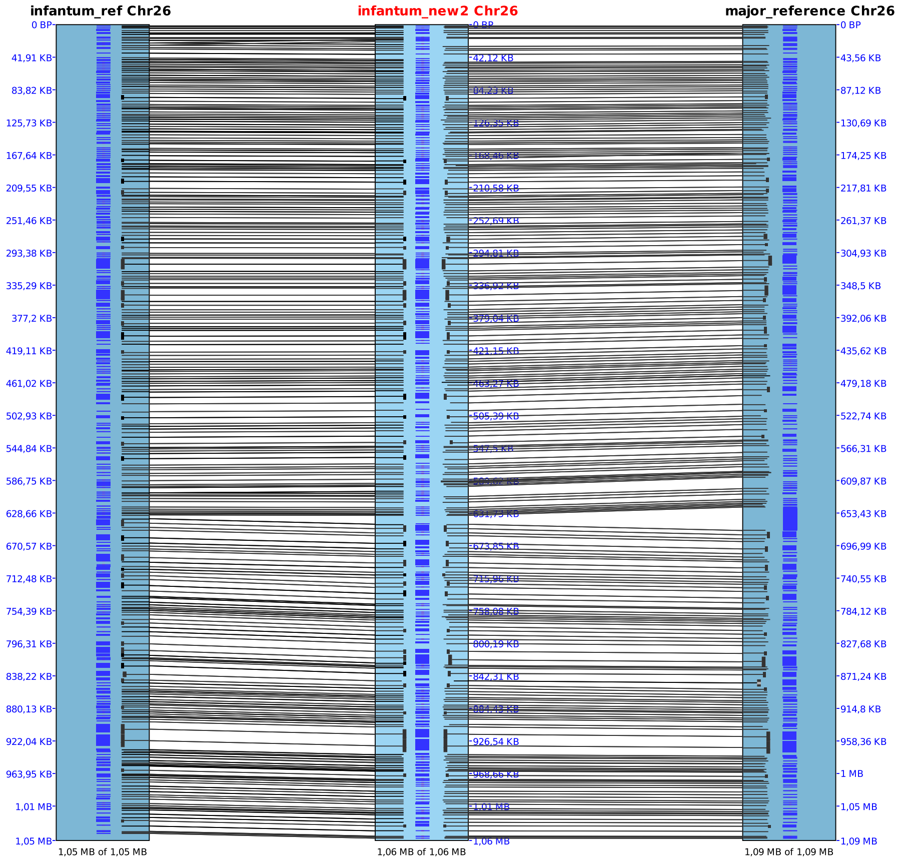
infantum_ref Chr26
infantum_new2 Chr26
major_reference Chr26
0 BP
41,91 KB
83,82 KB
125,73 KB
167,64 KB
209,55 KB
251,46 KB
293,38 KB
335,29 KB
377,2 KB
419,11 KB
461,02 KB
502,93 KB
544,84 KB
586,75 KB
628,66 KB
670,57 KB
712,48 KB
754,39 KB
796,31 KB
838,22 KB
880,13 KB
922,04 KB
963,95 KB
1,01 MB
1,05 MB
1,05 MB of 1,05 MB
0 BP
42,12 KB
84,23 KB
126,35 KB
168,46 KB
210,58 KB
252,69 KB
294,81 KB
336,92 KB
379,04 KB
421,15 KB
463,27 KB
505,39 KB
547,5 KB
631,73 KB
673,85 KB
715,96 KB
758,08 KB
800,19 KB
842,31 KB
884,42 KB
926,54 KB
968,66 KB
1,01 MB
1,06 MB
1,06 MB of 1,06 MB
0 BP
43,56 KB
87,12 KB
130,69 KB
174,25 KB
217,81 KB
261,37 KB
304,93 KB
348,5 KB
392,06 KB
435,62 KB
479,18 KB
522,74 KB
566,31 KB
609,87 KB
653,43 KB
696,99 KB
740,55 KB
784,12 KB
827,68 KB
871,24 KB
914,8 KB
958,36 KB
1 MB
1,05 MB
1,09 MB
1,09 MB of 1,09 MB

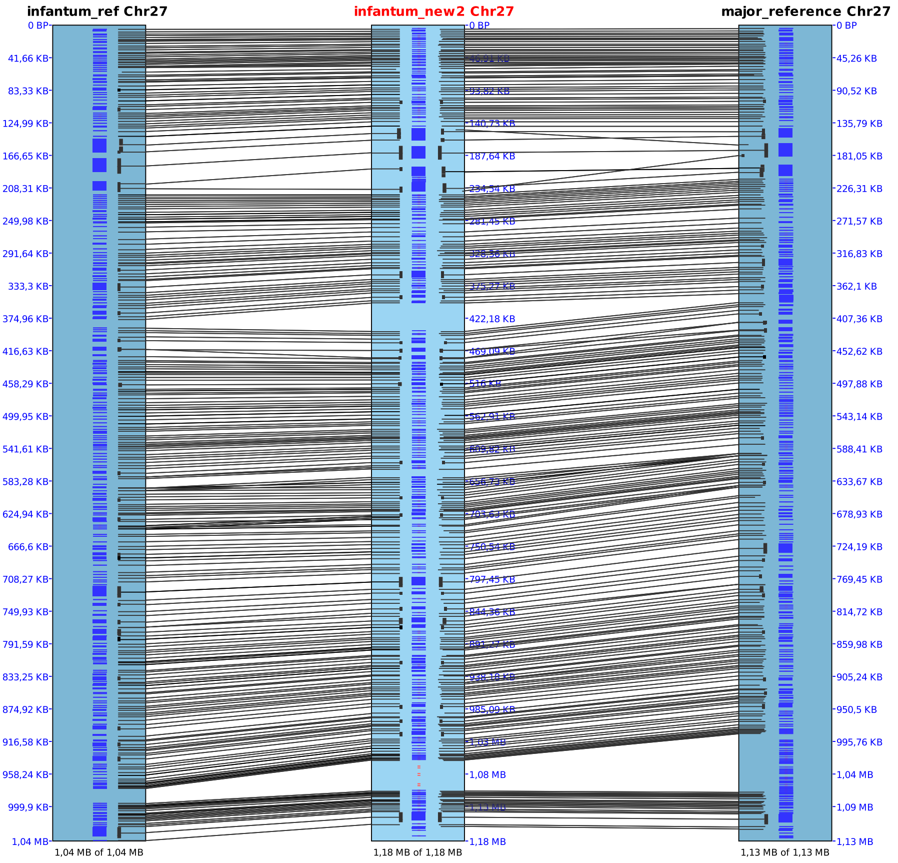

infantum_ref Chr27
infantum_new2 Chr27
major_reference Chr27
0 BP
41,66 KB
83,33 KB
124,99 KB
166,65 KB
208,31 KB
249,98 KB
291,64 KB
333,3 KB
374,96 KB
416,63 KB
458,29 KB
499,95 KB
541,61 KB
583,28 KB
624,94 KB
666,6 KB
708,27 KB
749,93 KB
791,59 KB
833,25 KB
874,92 KB
916,58 KB
958,24 KB
999,9 KB
1,04 MB
1,04 MB of 1,04 MB
0 BP
422,18 KB
1,08 MB
1,18 MB
1,18 MB of 1,18 MB
0 BP
45,26 KB
90,52 KB
135,79 KB
181,05 KB
226,31 KB
271,57 KB
316,83 KB
362,1 KB
407,36 KB
452,62 KB
497,88 KB
543,14 KB
588,41 KB
633,67 KB
678,93 KB
724,19 KB
769,45 KB
814,72 KB
859,98 KB
905,24 KB
950,5 KB
995,76 KB
1,04 MB
1,09 MB
1,13 MB
1,13 MB of 1,13 MB

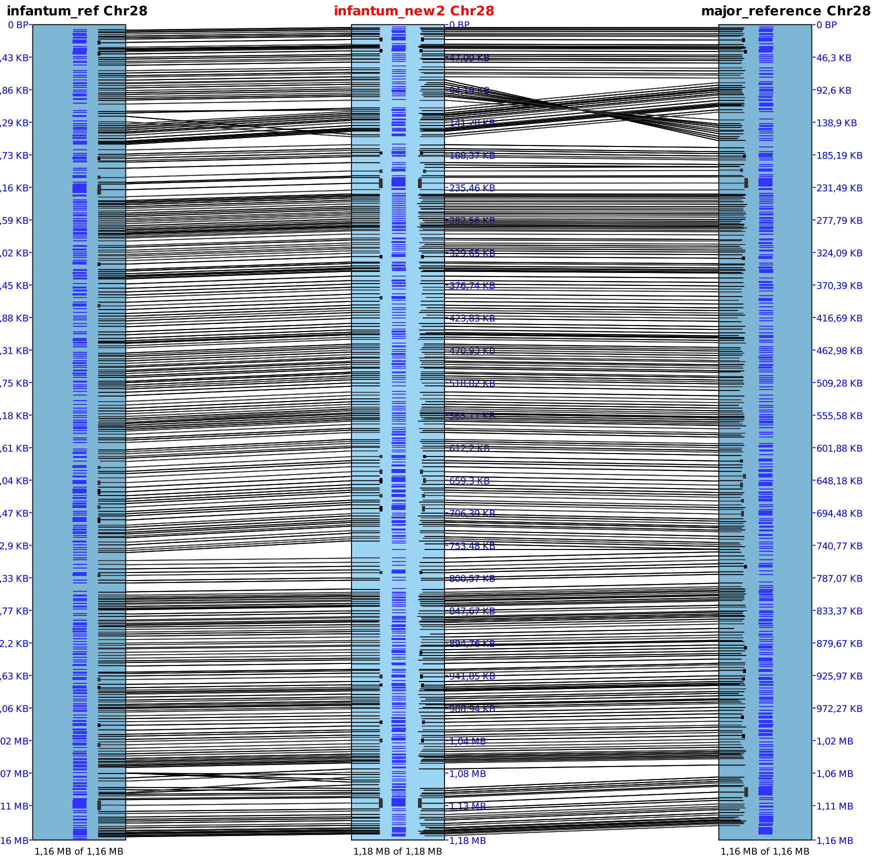
infantum_ref Chr28
infantum_new2 Chr28
major_reference Chr28
0 BP
46,43 KB
92,86 KB
139,29 KB
185,73 KB
232,16 KB
278,59 KB
325,02 KB
371,45 KB
417,88 KB
464,31 KB
510,75 KB
557,18 KB
603,61 KB
650,04 KB
696,47 KB
742,9 KB
789,33 KB
835,77 KB
882,2 KB
928,63 KB
975,06 KB
1,02 MB
1,07 MB
1,11 MB
1,16 MB
1,16 MB of 1,16 MB
0 BP
47,09 KB
94,19 KB
141,28 KB
188,37 KB
235,46 KB
282,56 KB
329,65 KB
376,74 KB
423,83 KB
470,93 KB
518,02 KB
565,11 KB
612,2 KB
659,3 KB
706,39 KB
753,48 KB
800,57 KB
847,67 KB
894,76 KB
941,85 KB
988,94 KB
1,04 MB
1,08 MB
1,13 MB
1,18 MB
1,18 MB of 1,18 MB
0 BP
46,3 KB
92,6 KB
138,9 KB
185,19 KB
231,49 KB
277,79 KB
324,09 KB
370,39 KB
416,69 KB
462,98 KB
509,28 KB
555,58 KB
601,88 KB
648,18 KB
694,48 KB
740,77 KB
787,07 KB
833,37 KB
879,67 KB
925,97 KB
972,27 KB
1,02 MB
1,06 MB
1,11 MB
1,16 MB
1,16 MB of 1,16 MB

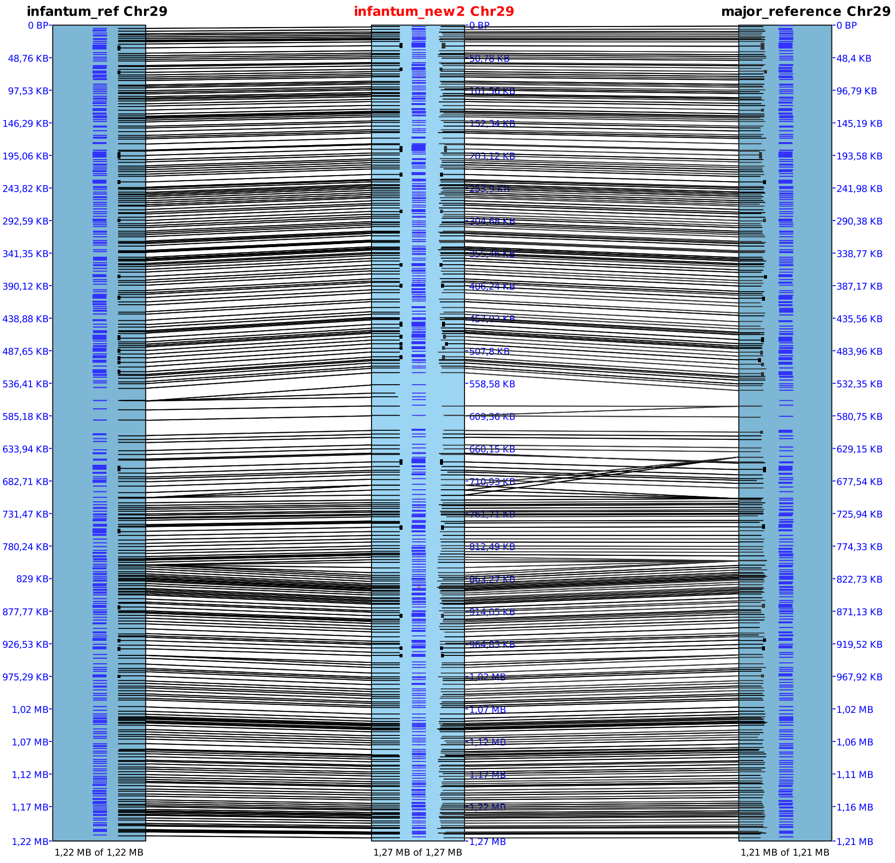
infantum_ref Chr29
infantum_new2 Chr29
major_reference Chr29
0 BP
48,76 KB
97,53 KB
146,29 KB
195,06 KB
243,82 KB
292,59 KB
341,35 KB
390,12 KB
438,88 KB
487,65 KB
536,41 KB
585,18 KB
633,94 KB
682,71 KB
731,47 KB
780,24 KB
829 KB
877,77 KB
926,53 KB
975,29 KB
1,02 MB
1,07 MB
1,12 MB
1,17 MB
1,22 MB
1,22 MB of 1,22 MB
0 BP
50,78 KB
101,56 KB
152,34 KB
203,12 KB
253,9 KB
304,68 KB
355,46 KB
406,24 KB
457,02 KB
507,8 KB
558,58 KB
609,36 KB
660,15 KB
710,93 KB
761,71 KB
812,49 KB
863,27 KB
914,05 KB
964,83 KB
1,02 MB
1,07 MB
1,12 MB
1,17 MB
1,22 MB
1,27 MB
1,27 MB of 1,27 MB
0 BP
48,4 KB
96,79 KB
145,19 KB
193,58 KB
241,98 KB
290,38 KB
338,77 KB
387,17 KB
435,56 KB
483,96 KB
532,35 KB
580,75 KB
629,15 KB
677,54 KB
725,94 KB
774,33 KB
822,73 KB
871,13 KB
919,52 KB
967,92 KB
1,02 MB
1,06 MB
1,11 MB
1,16 MB
1,21 MB
1,21 MB of 1,21 MB

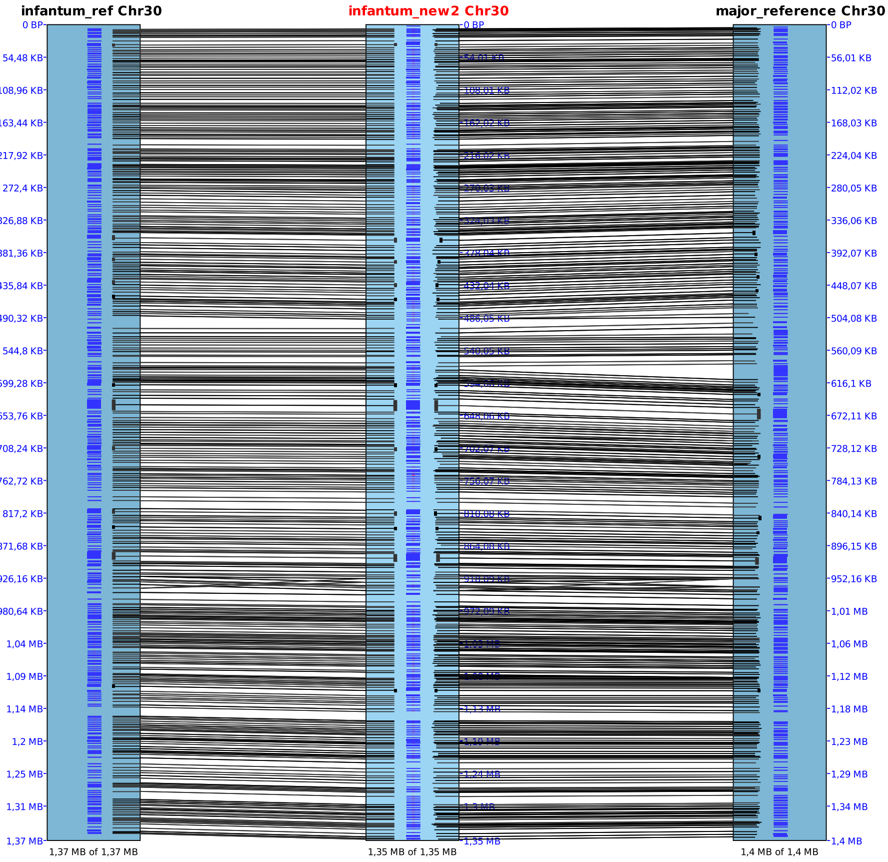
infantum_ref Chr30
infantum_new2 Chr30
major_reference Chr30
0 BP
54,48 KB
108,96 KB
163,44 KB
217,92 KB
272,4 KB
326,88 KB
381,36 KB
435,84 KB
490,32 KB
544,8 KB
599,28 KB
653,76 KB
708,24 KB
762,72 KB
817,2 KB
871,68 KB
926,16 KB
980,64 KB
1,04 MB
1,09 MB
1,14 MB
1,2 MB
1,25 MB
1,31 MB
1,37 MB
1,37 MB of 1,37 MB
0 BP
54,01 KB
108,01 KB
162,02 KB
216,02 KB
270,03 KB
324,03 KB
378,04 KB
432,04 KB
486,05 KB
540,05 KB
648,06 KB
702,07 KB
756,07 KB
810,08 KB
864,08 KB
918,09 KB
972,09 KB
1,13 MB
1,24 MB
1,3 MB
1,35 MB
1,35 MB of 1,35 MB
0 BP
56,01 KB
112,02 KB
168,03 KB
224,04 KB
280,05 KB
336,06 KB
392,07 KB
448,07 KB
504,08 KB
560,09 KB
616,1 KB
672,11 KB
728,12 KB
784,13 KB
840,14 KB
896,15 KB
952,16 KB
1,01 MB
1,06 MB
1,12 MB
1,18 MB
1,23 MB
1,29 MB
1,34 MB
1,4 MB
1,4 MB of 1,4 MB

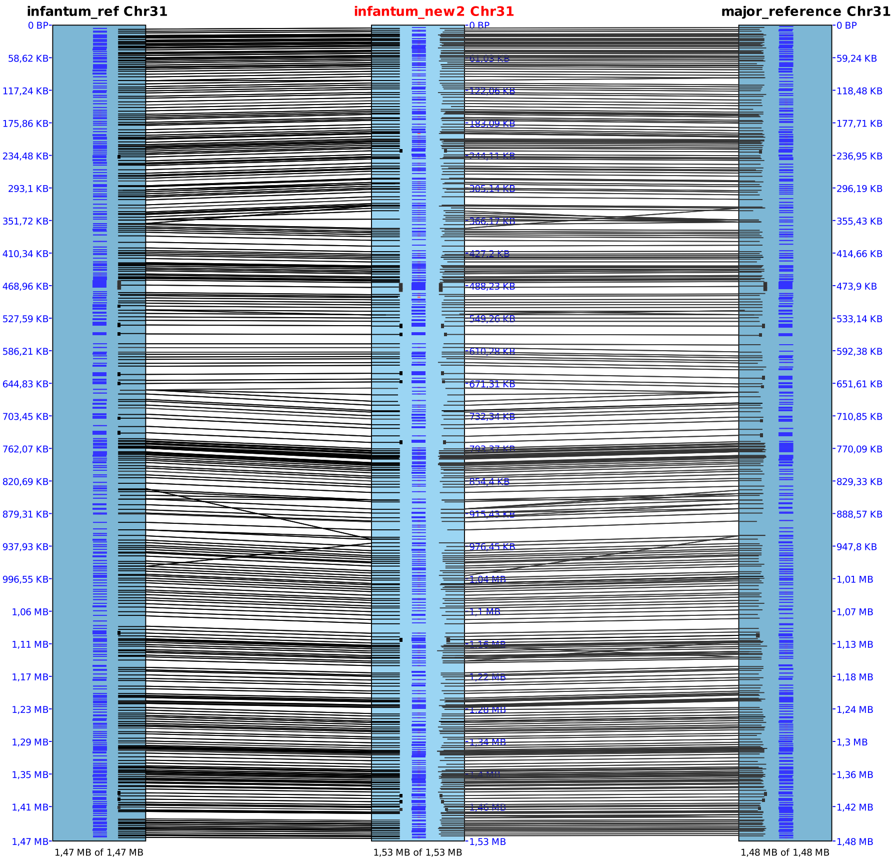

infantum_ref Chr31
infantum_new2 Chr31
major_reference Chr31
0 BP
58,62 KB
117,24 KB
175,86 KB
234,48 KB
293,1 KB
351,72 KB
410,34 KB
468,96 KB
527,59 KB
586,21 KB
644,83 KB
703,45 KB
762,07 KB
820,69 KB
879,31 KB
937,93 KB
996,55 KB
1,06 MB
1,11 MB
1,17 MB
1,23 MB
1,29 MB
1,35 MB
1,41 MB
1,47 MB
1,47 MB of 1,47 MB
0 BP
61,03 KB
122,06 KB
183,09 KB
244,11 KB
305,14 KB
366,17 KB
427,2 KB
488,23 KB
549,26 KB
610,28 KB
671,31 KB
732,34 KB
793,37 KB
854,4 KB
915,43 KB
976,45 KB
1,04 MB
1,1 MB
1,16 MB
1,22 MB
1,28 MB
1,34 MB
1,4 MB
1,46 MB
1,53 MB
1,53 MB of 1,53 MB
0 BP
59,24 KB
118,48 KB
177,71 KB
236,95 KB
296,19 KB
355,43 KB
414,66 KB
473,9 KB
533,14 KB
592,38 KB
651,61 KB
710,85 KB
770,09 KB
829,33 KB
888,57 KB
947,8 KB
1,01 MB
1,07 MB
1,13 MB
1,18 MB
1,24 MB
1,3 MB
1,36 MB
1,42 MB
1,48 MB
1,48 MB of 1,48 MB

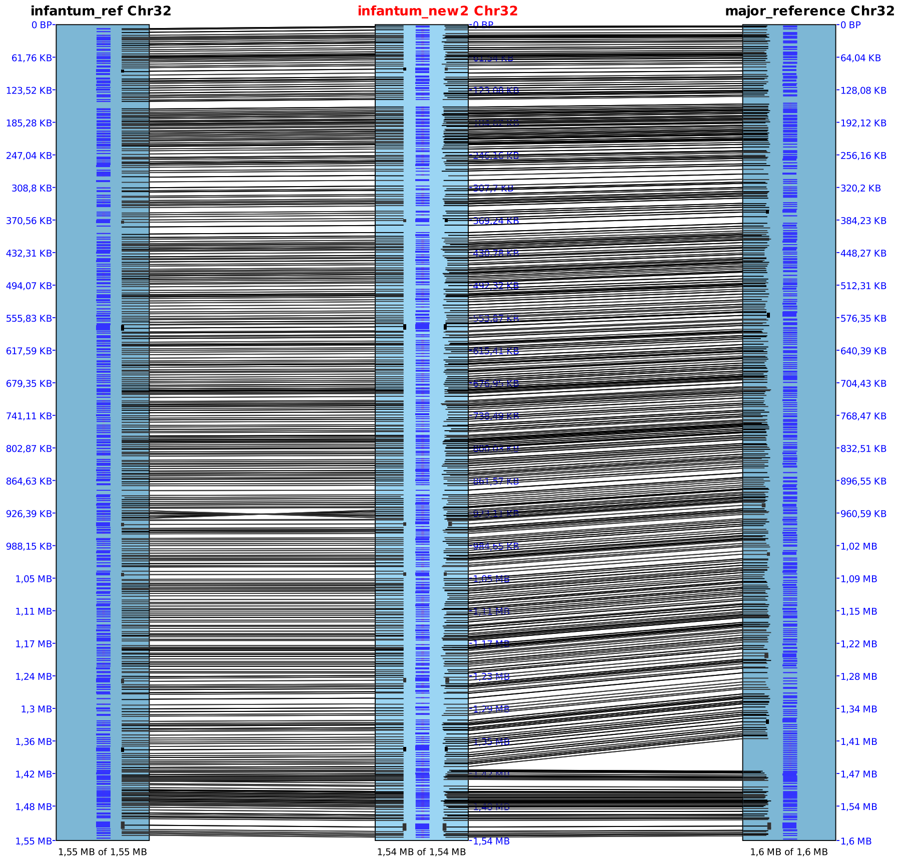
infantum_ref Chr32
infantum_new2 Chr32
major_reference Chr32
0 BP
61,76 KB
123,52 KB
185,28 KB
247,04 KB
308,8 KB
370,56 KB
432,31 KB
494,07 KB
555,83 KB
617,59 KB
679,35 KB
741,11 KB
802,87 KB
864,63 KB
926,39 KB
988,15 KB
1,05 MB
1,11 MB
1,17 MB
1,24 MB
1,3 MB
1,36 MB
1,42 MB
1,48 MB
1,55 MB
0 BP
1,05 MB
1,54 MB
0 BP
64,04 KB
128,08 KB
192,12 KB
256,16 KB
320,2 KB
384,23 KB
448,27 KB
512,31 KB
576,35 KB
640,39 KB
704,43 KB
768,47 KB
832,51 KB
896,55 KB
960,59 KB
1,02 MB
1,09 MB
1,15 MB
1,22 MB
1,28 MB
1,34 MB
1,41 MB
1,47 MB
1,54 MB
1,6 MB
1,55 MB of 1,55 MB
1,54 MB of 1,54 MB
1,6 MB of 1,6 MB

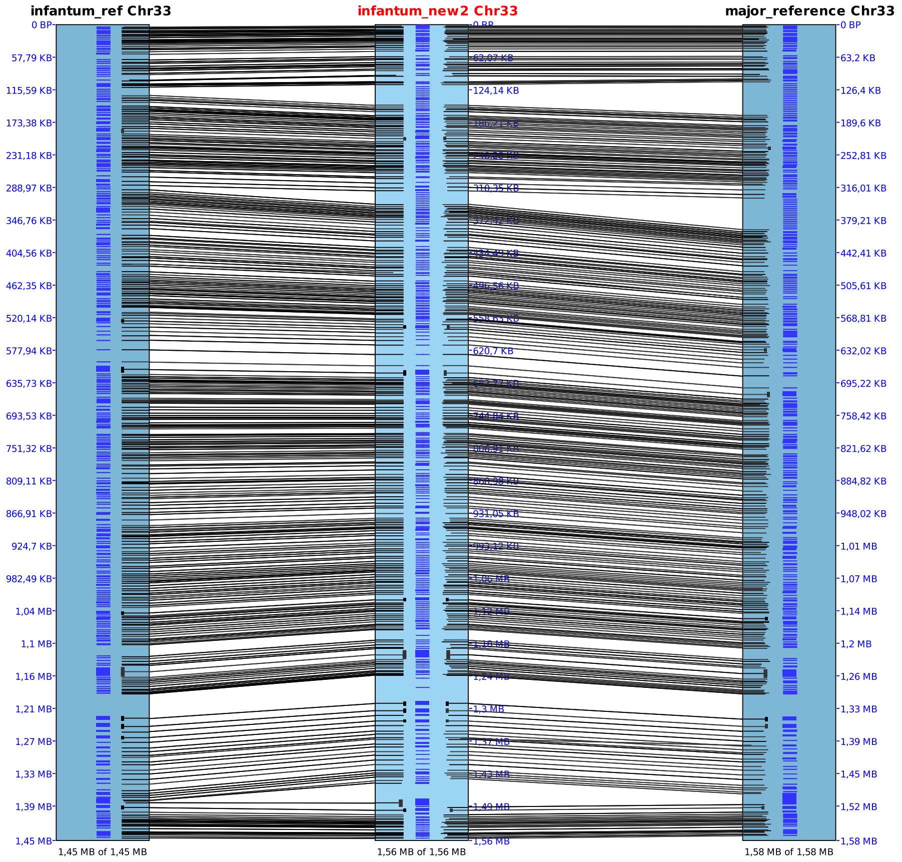

infantum_ref Chr33
infantum_new2 Chr33
major_reference Chr33
0 BP
57,79 KB
115,59 KB
173,38 KB
231,18 KB
288,97 KB
346,76 KB
404,56 KB
462,35 KB
520,14 KB
577,94 KB
635,73 KB
693,53 KB
751,32 KB
809,11 KB
866,91 KB
924,7 KB
982,49 KB
1,04 MB
1,1 MB
1,16 MB
1,21 MB
1,27 MB
1,33 MB
1,39 MB
1,45 MB
1,45 MB of 1,45 MB
0 BP
62,07 KB
124,14 KB
310,35 KB
620,7 KB
931,05 KB
1,3 MB
1,49 MB
1,56 MB
1,56 MB of 1,56 MB
0 BP
63,2 KB
126,4 KB
189,6 KB
252,81 KB
316,01 KB
379,21 KB
442,41 KB
505,61 KB
568,81 KB
632,02 KB
695,22 KB
758,42 KB
821,62 KB
884,82 KB
948,02 KB
1,01 MB
1,07 MB
1,14 MB
1,2 MB
1,26 MB
1,33 MB
1,39 MB
1,45 MB
1,52 MB
1,58 MB
1,58 MB of 1,58 MB

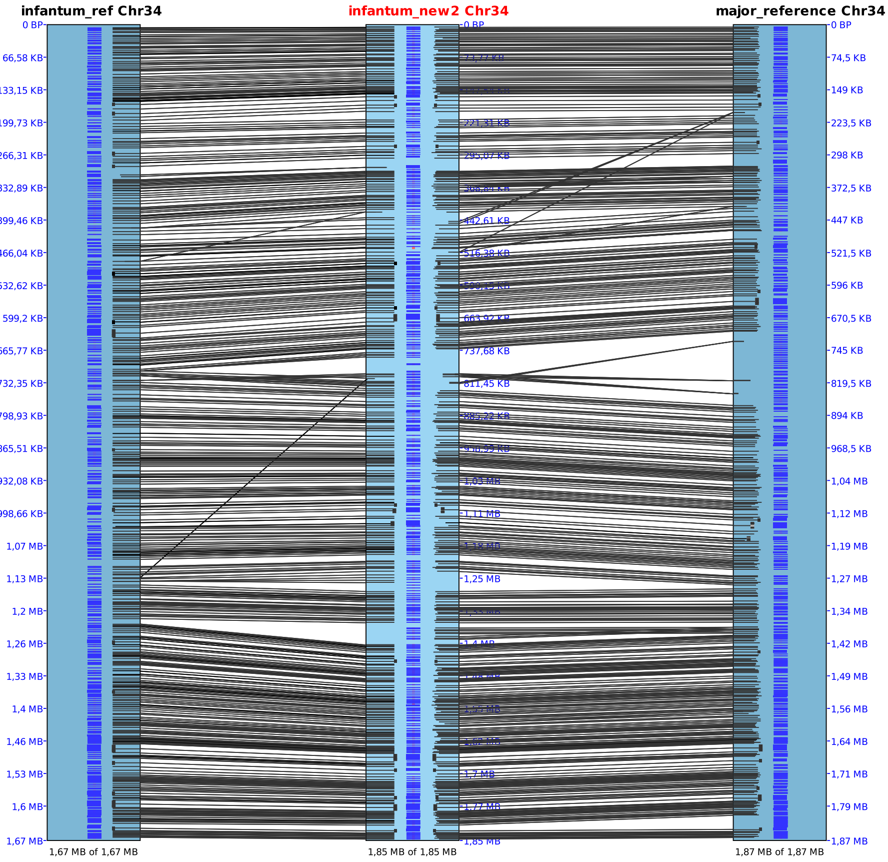
infantum_ref Chr34
infantum_new2 Chr34
major_reference Chr34
0 BP
66,58 KB
133,15 KB
199,73 KB
266,31 KB
332,89 KB
399,46 KB
466,04 KB
532,62 KB
599,2 KB
665,77 KB
732,35 KB
798,93 KB
865,51 KB
932,08 KB
998,66 KB
1,07 MB
1,13 MB
1,2 MB
1,26 MB
1,33 MB
1,4 MB
1,46 MB
1,53 MB
1,6 MB
1,67 MB
1,67 MB of 1,67 MB
0 BP
221,31 KB
295,07 KB
442,61 KB
516,38 KB
663,92 KB
737,68 KB
811,45 KB
1,03 MB
1,11 MB
1,18 MB
1,25 MB
1,4 MB
1,7 MB
1,77 MB
1,85 MB
1,85 MB of 1,85 MB
0 BP
74,5 KB
149 KB
223,5 KB
298 KB
372,5 KB
447 KB
521,5 KB
596 KB
670,5 KB
745 KB
819,5 KB
894 KB
968,5 KB
1,04 MB
1,12 MB
1,19 MB
1,27 MB
1,34 MB
1,42 MB
1,49 MB
1,56 MB
1,64 MB
1,71 MB
1,79 MB
1,87 MB
1,87 MB of 1,87 MB

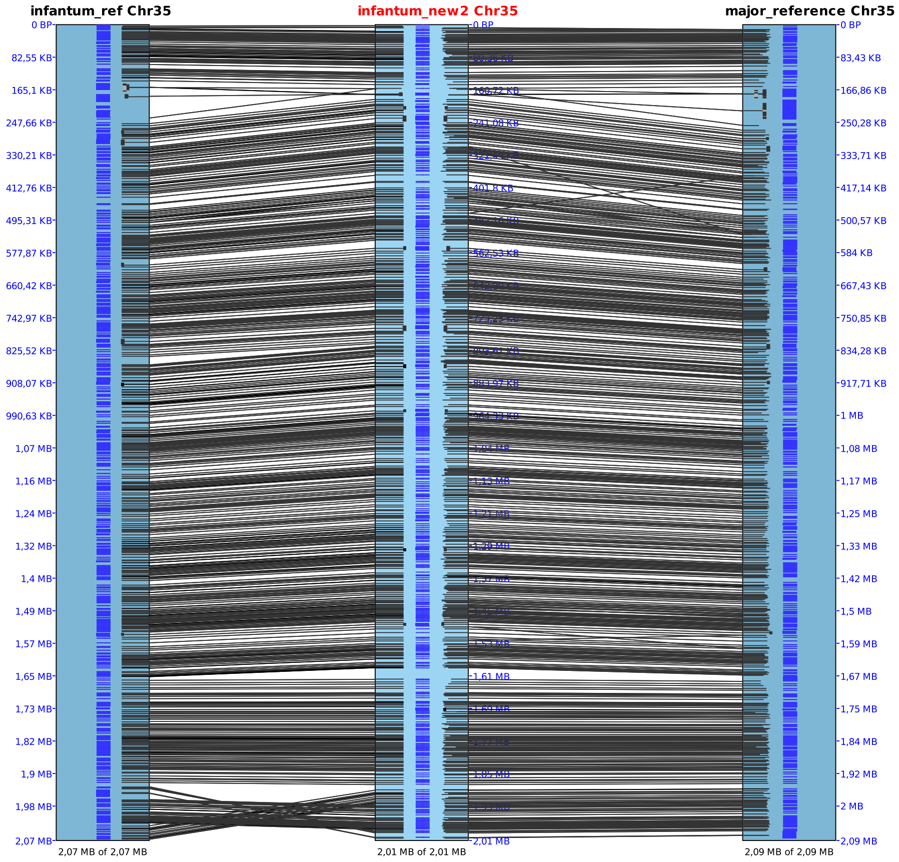
infantum_ref Chr35
infantum_new2 Chr35
major_reference Chr35
0 BP
82,55 KB
165,1 KB
247,66 KB
330,21 KB
412,76 KB
495,31 KB
577,87 KB
660,42 KB
742,97 KB
825,52 KB
908,07 KB
990,63 KB
1,07 MB
1,16 MB
1,24 MB
1,32 MB
1,4 MB
1,49 MB
1,57 MB
1,65 MB
1,73 MB
1,82 MB
1,9 MB
1,98 MB
2,07 MB
2,07 MB of 2,07 MB
0 BP
160,72 KB
241,08 KB
401,8 KB
562,53 KB
883,97 KB
964,33 KB
1,04 MB
1,13 MB
1,21 MB
1,29 MB
1,37 MB
1,53 MB
1,61 MB
1,69 MB
1,77 MB
1,85 MB
2,01 MB
2,01 MB of 2,01 MB
0 BP
83,43 KB
166,86 KB
250,28 KB
333,71 KB
417,14 KB
500,57 KB
584 KB
667,43 KB
750,85 KB
834,28 KB
917,71 KB
1 MB
1,08 MB
1,17 MB
1,25 MB
1,33 MB
1,42 MB
1,5 MB
1,59 MB
1,67 MB
1,75 MB
1,84 MB
1,92 MB
2 MB
2,09 MB
2,09 MB of 2,09 MB

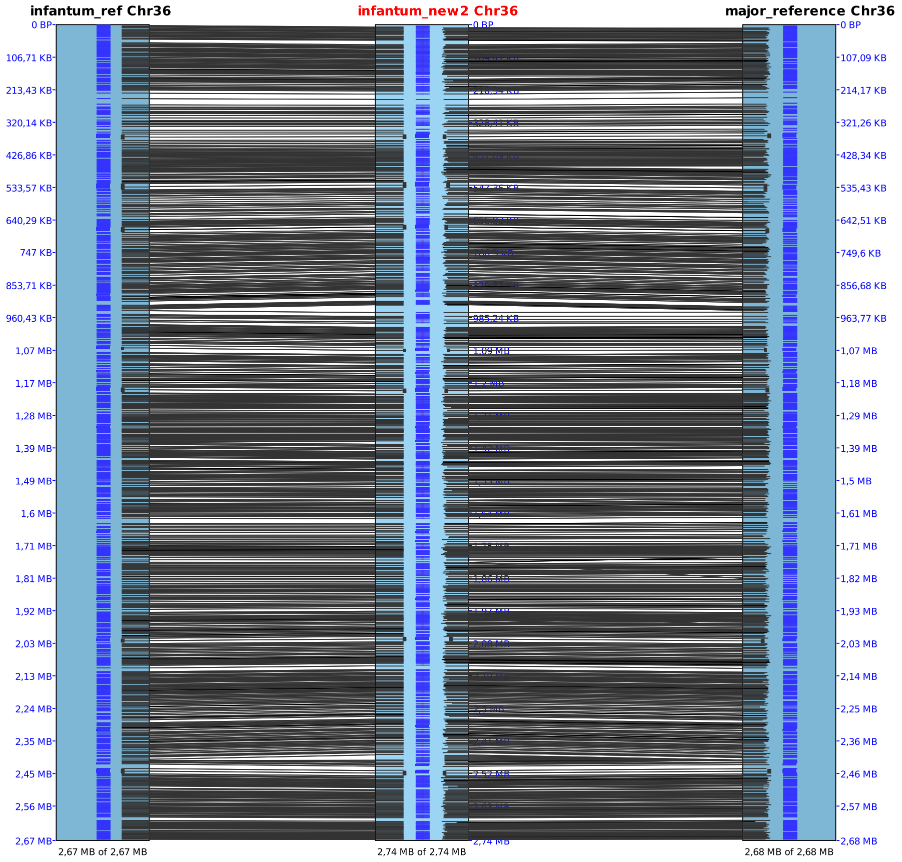
infantum_ref Chr36
infantum_new2 Chr36
major_reference Chr36
0 BP
106,71 KB
213,43 KB
320,14 KB
426,86 KB
533,57 KB
640,29 KB
747 KB
853,71 KB
960,43 KB
1,07 MB
1,17 MB
1,28 MB
1,39 MB
1,49 MB
1,6 MB
1,71 MB
1,81 MB
1,92 MB
2,03 MB
2,13 MB
2,24 MB
2,35 MB
2,45 MB
2,56 MB
2,67 MB
2,67 MB of 2,67 MB
0 BP
218,94 KB
328,41 KB
547,36 KB
985,24 KB
1,09 MB
1,86 MB
2,08 MB
2,52 MB
2,74 MB
2,74 MB of 2,74 MB
0 BP
107,09 KB
214,17 KB
321,26 KB
428,34 KB
535,43 KB
642,51 KB
749,6 KB
856,68 KB
963,77 KB
1,07 MB
1,18 MB
1,29 MB
1,39 MB
1,5 MB
1,61 MB
1,71 MB
1,82 MB
1,93 MB
2,03 MB
2,14 MB
2,25 MB
2,36 MB
2,46 MB
2,57 MB
2,68 MB
2,68 MB of 2,68 MB
